# Fractal fractional model for tuberculosis: existence and numerical solutions

**DOI:** 10.1038/s41598-024-62386-4

**Published:** 2024-05-28

**Authors:** Aziz Khan, Kamal Shah, Thabet Abdeljawad, Inas Amacha

**Affiliations:** 1 Department of Mathematics and Sciences, Prince Sultan University, P.O. Box 66833, 11586 Riyadh, Saudi Arabia; 2 Department of Medical Research, China Medical University, Taichung, 40402 Taiwan

**Keywords:** Fractal fractional operator, UH stability, Existence criteria, Numerical analysis, Banach’s contraction, 26A33, 34A08, 34A12, Computational models, Mathematics and computing

## Abstract

This paper deals with the mathematical analysis of Tuberculosis by using fractal fractional operator. Mycobacterium TB is the bacteria that causes tuberculosis. This airborne illness mostly impacts the lungs but may extend to other body organs. When the infected individual coughs, sneezes or speaks, the bacterium gets released into the air and travels from one person to another. Five classes have been formulated to study the dynamics of this disease: susceptible class, infected of DS, infected of MDR, isolated class, and recovered class. To study the suggested fractal fractional model’s wellposedness associated with existence results, and boundedness of solutions. Further, the invariant region of the considered model, positive solutions, equilibrium point, and reproduction number. One would typically employ a fractional calculus approach to obtain numerical solutions for the fractional order Tuberculosis model using the Adams-Bashforth-Moulton method. The fractional order derivatives in the model can be approximated using appropriate numerical schemes designed for fractional order differential equations.

## Introduction

Tuberculosis (TB) is a bacterial infection caused by Mycobacterium tuberculosis. It typically affects the lungs, but it can also affect other organs such as the spine, brain, and kidneys. When an infected person talks, sneezes, or coughs, bacteria-containing droplets are released into the air and ingested by others. When an infected individual coughs, sneezes, or talks, the bacteria-containing droplets are breathed by others. Tuberculosis can proliferate and cause an infection once inside the body. However, not everyone who is infected with tuberculosis develops active illness. The immune system is usually able to suppress the infection, resulting in a latent TB infection^1^. People with latent tuberculosis do not have symptoms and are not infectious, but the germs can remain dormant in their bodies and become active later if the immune system is compromised. Global TB Burden: The World Health Organization (WHO) estimates that 10 million individuals worldwide will contract tuberculosis (TB) by 2020. Deaths from tuberculosis: Approximately 1.5 million people died from tuberculosis in 2020, see details in^2–4^. It is critical to remember that many deaths are avoidable with early discovery and treatment. Co-infection of tuberculosis and HIV: Tuberculosis is the primary cause of mortality in persons living with HIV/AIDS. Around 208,000 HIV-positive people died from tuberculosis in 2020. TB is prevalent in every country, although the burden is greatest in low- and middle-income countries. More than 95 percent of TB infections and fatalities in 2020 occurred in these settings. High-Burden Countries: China, India, Indonesia, Philippines, Nigeria, Pakistan, Bangladesh, South Africa, the Democratic Republic of Ethiopia and Congo, had the largest number of TB cases in 2020^7–10^. TB is diagnosed using a variety of techniques, including the tuberculin skin test, interferon-gamma release assays, chest X-rays, and sputum culture. To guarantee total eradication of the germs, TB treatment typically consists of a mixture of medicines administered over several months. Isoniazid, rifampin, ethambutol, and pyrazinamide are the most regularly used antibiotics for tuberculosis therapy. The World Health Organisation (WHO) and various other organizations play an important role in eliminating the spread of tuberculosis (TB). These efforts enhance the identification of initial symptoms access to top-notch care, and medication, advance new vaccines, and diagnostic investigation see in^5,6^. Tuberculosis continues a serious public health challenge despite even the advancements in technology, especially in those located in low and middle income countries. International global coordination is required to stop the spreading TB.

**Figure 1:**
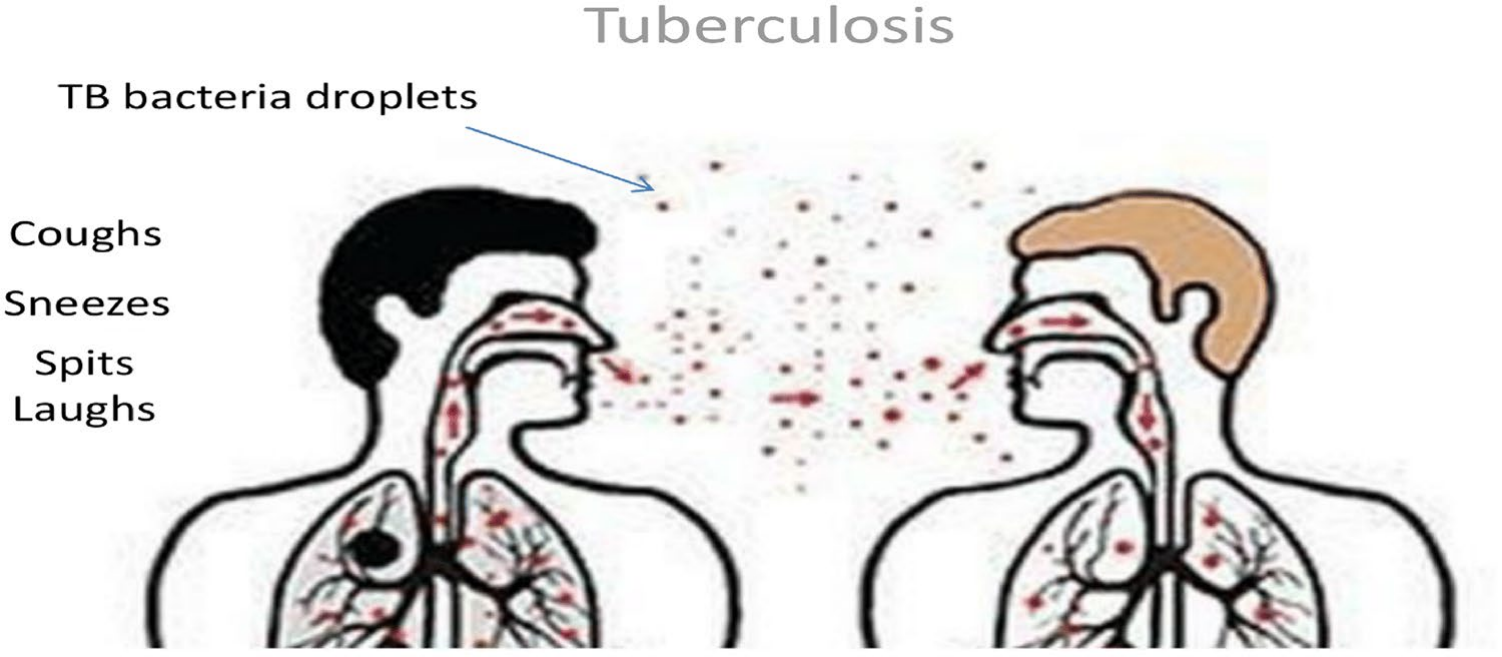
Tuberculosis spreading.

Mathematical modeling plays a crucial role in investigating infectious diseases. This method is valuable for analyzing the patterns and dynamics of infectious diseases like HIV, TB, AIDS, and influenza virus, see details in^11^–^25^. By incorporating variables such as disease characteristics, population rates, and connection rates, Mathematical models can portray their interconnection of susceptibles, infected, and recovered members of the diseases. These models closely predict and forecast the onset of diseases, and evaluate, the efficacy of strategies, such as medication, quarantine, vaccination, and isolation based on data availability. Mathematical frameworks can be designed for various purposes such as medical access, demographic changes, and staging rates of diseases. These strategies help control or eliminate diseases, one can see^7,8^. With the use of mathematics-based modeling, researchers can examine a broad range of scenarios and assess the possible outcomes of different tactics, such as building more hospitals or enacting social distancing measures, which aids in the early detection and response to epidemics. Computational frameworks powered by real-time data can modify and deliver updated estimates, which could guide public health programs and impact policy decisions.

Mathematical models of fractal fractions provide an intriguing and advanced framework for examining intricate and self-repeating variations in a variety of systems. In contrast to standard mathematical models, that frequently rely on integer dimensions as well as smooth curves, fractal fractional models embrace the idea of fractional dimensions, allowing for the portrayal of complicated patterns observed in nature, art, and even financial markets. Khan et al.^26^ investigated the existence criterion, numerical analysis, and stability for the six classes of the fractal fractional TB model. Farman et al.^27^ studied a fractional-order model for HIV/AIDS that includes an antiretroviral therapy compartment. The Sumudu transform is utilized in the study to improve the dependability of the results in an advanced way. Bonyah et al.^28^ A listeriosis mathematical model integrates fractal-fractional ordering utilizing Caputo and Atangana-Baleanu derivatives, allowing researchers to investigate the disease’s future behaviors with memory effects. The steady states and the infection spread threshold parameters are calculated, with numerical results presented for each fractal-fractional-order operator. Partohaghighi et al.^29^ presented a fractal-fractional computer virus system, and use the Atangana-Baleanu operator. The paper verifies uniqueness using fixed-point theorems, leverages Atangana-Toufik approaches for approximate solutions and uses visual illustrations to analyze the method’s effectiveness with different orders and beginning circumstances. Singh et al.^30^ investigated the dynamics of a fish farm model with arbitrary order Atangana-Baleanu derivatives, which includes nutrients, fish, and mussels, as well as discrete gestational delays in fish reproduction. To find results and examine the influence of the non-integer order derivative on nutrients, fish, and mussel populations, by employing the Homotopy Analysis Transforms Method (HATM). The Picard-Lindelof technique is used to check out the existence as well as the unique solutions. Makhlouf and Baleanu^31^ studied the Finite-Time Stability of Neutral Fractional Order Systems with Time Delay. Unlike prior techniques that relied on the Gronwall inequality, we employ fixed point theory to build Finite-Time Stability. To confirm and reinforce the theoretical contributions, two examples are offered. Baleanu et al.^32^ investigate the multidisciplinary phases of nonlinear research, intensifying the difficulties in inventive complicated processes and presuming for systematic mathematical frameworks. It looks into how to employ neural networks and FODEs for chaotic and nonlinear systems. Innovative works from a class of domains on fractals, chaos, fractional calculus, differential equations, and machine learning are involved in this special issue to present enhanced comprehension and models for complected mathematical problems. Models of fractal fractional diseases give a new approach to studying the dynamics of disease and its transmission throughout populations. These models, which are influenced by fractional calculus and fractal geometry, provide insight into the complex and interconnected structure of disease propagation. These models also take into account the concept of fractional calculus, which explains the fractional behavior of disease spreading using non-integer order. A more precise portrayal of long-term memory and dependence on disease dynamics is provided by this fractional approach. Fractal fractional disease simulations can better capture the persistence and recurring dynamics seen in infectious diseases by involving memory significance and non-Markovian behavior. One can study in detail therein^33^–^44^.

## Model formulation

**Figure 2:**
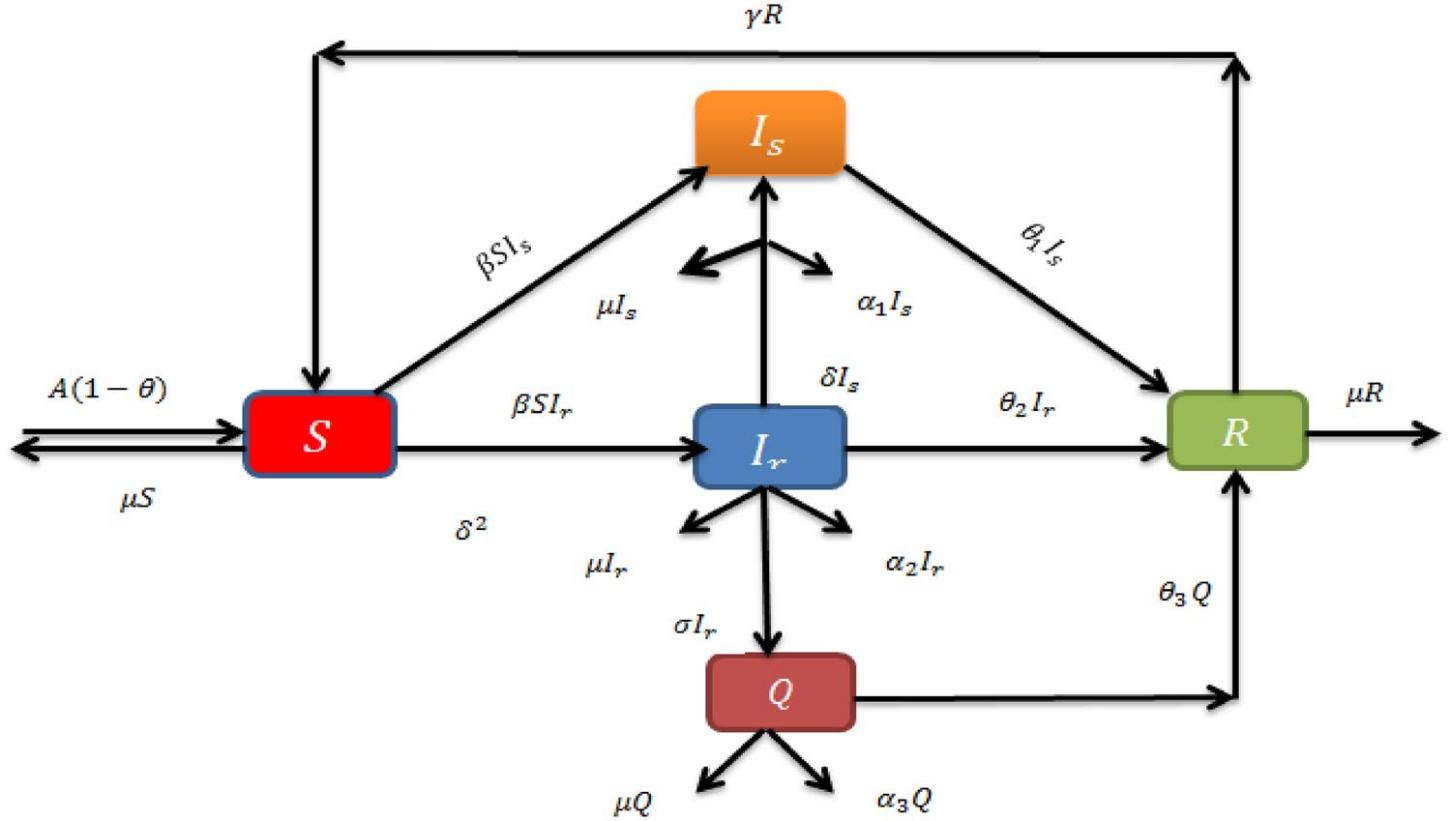
Flow chart of TB mathematical model.

We’ll look at Bhadauria et al.^10^ deterministic mathematical model for TB, which includes drug-sensitive (DS), multidrug-resistant (MDR) classes in the model. Tuberculosis is spreading in different ways one can see, in Fig. 1. Therefore it is classified in five classes and their connection with each other in Fig. 2, where ${\mathcal {S}}:$ Susceptible class, ${\mathcal {I}}_{s}:$ DS infected class, ${\mathcal {I}}_{s}:$ MDR infected class, ${\mathcal {Q}}:$ Isolated class, ${\mathcal {R}}:$ recovered class. The transformation ratios are stand for $\Theta _{1}:$ rate of DS, $\theta _{2}$ rate of MDR, $\theta _{3}:$ rate of isolation, $\beta :$ rate of TB incidence, ${\mathcal {A}}:$ rate of recruitment, $\theta :$ rate of vaccination, $\xi$ transmission from ${\mathcal {I}}_{s}$ to ${\mathcal {I}}_{r}$,$\mu :$ rate of natural normality, $\gamma :$ rate of recovery, $\alpha _{1}:$ rate death from ${\mathcal {I}}_{s}$, ${\alpha }_{2}$ death rate from ${\mathcal {I}}_{r}$, ${\alpha }_{3}$ death rate from ${\mathcal {Q}}$ class

1 $$\begin{aligned} {\left\{ \begin{array}{ll} \textbf{D}_{t}{\mathcal {S}}=(1-\theta ){\mathcal {A}}-\beta {\mathcal {I}}_{s}{\mathcal {S}} -\beta {\mathcal {I}}_{r}{\mathcal {S}}-\mu {\mathcal {S}}+\gamma {\mathcal {R}}\\ \textbf{D}_{t}{\mathcal {I}}_{s}=\beta {\mathcal {I}}_{s}{\mathcal {S}}-(\mu +\theta _{1} +\xi +\alpha _{1}){\mathcal {I}}_{s}\\ \textbf{D}_{t}{\mathcal {I}}_{r}=\beta {\mathcal {I}}_{r}{\mathcal {S}}-(\mu +\theta _{2} +\xi +\alpha _{2}){\mathcal {I}}_{r}+\xi {\mathcal {I}}_{s}\\ \textbf{D}_{t}{\mathcal {Q}}=\sigma {\mathcal {I}}_{r}-(\mu +\theta _{3}+\alpha _{3}) {\mathcal {Q}}+\sigma {\mathcal {I}}_{r}\\ \textbf{D}_{t}{\mathcal {R}}=\theta _{1}{\mathcal {I}}_{s}+\theta _{2}{\mathcal {I}}_{r} +\theta _{3}{\mathcal {Q}}-(\mu +\gamma ){\mathcal {R}}.\\ \end{array}\right. } \end{aligned}$$

For the simplicity

$$\begin{aligned} \hbar 1=(\mu +\theta _{1}+\xi +\alpha _{1}), \hbar 2=(\mu +\theta _{2}+\xi +\alpha _{2}), \hbar 3=(\mu +\theta _{3}+\alpha _{3}), \end{aligned}$$

we can write the system of equation Eq. (1) in the following Eq. (2) form

2 $$\begin{aligned} {\left\{ \begin{array}{ll} \textbf{D}_{t}{\mathcal {S}}=(1-\theta ){\mathcal {A}}-\beta {\mathcal {I}}_{s}{\mathcal {S}} -\beta {\mathcal {I}}_{r}{\mathcal {S}}-\mu {\mathcal {S}}+\gamma {\mathcal {R}}\\ \textbf{D}_{t}{\mathcal {I}}_{s}=\beta {\mathcal {I}}_{s}{\mathcal {S}}-\hbar _{1}{\mathcal {I}}_{s}\\ \textbf{D}_{t}{\mathcal {I}}_{r}=\beta {\mathcal {I}}_{r}{\mathcal {S}}-\hbar _{2}{\mathcal {I}}_{r} +\xi {\mathcal {I}}_{s}\\ \textbf{D}_{t}{\mathcal {Q}}=\sigma {\mathcal {I}}_{r}-\hbar _{3}{\mathcal {Q}}+\sigma {\mathcal {I}}_{r}\\ \textbf{D}_{t}{\mathcal {R}}=\theta _{1}{\mathcal {I}}_{s}+\theta _{2}{\mathcal {I}}_{r}+\theta _{3} {\mathcal {Q}}-(\mu +\gamma ){\mathcal {R}}. \end{array}\right. } \end{aligned}$$

Subjected to initial conditions

3 $$\begin{aligned} ({\mathcal {S}},{\mathcal {I}}_{s},{\mathcal {I}}_{r},{\mathcal {Q}},{\mathcal {R}})\ge 0, \end{aligned}$$

Here we study a mathematical model for TB, which includes a DS and MDR infected classes in scheme with the following assumptions,

1. Susceptible Population: The model starts with a pool of susceptible individuals in the population ${\mathcal {S}}$, who are at risk of contracting tuberculosis. These people join the system at a rate ${\mathcal {A}}(1 -\theta )$, where ${\mathcal {A}}$, is the recruiting rate and is the vaccination rate. It is considered that immunisation gives complete protection against tuberculosis.
2. $\beta$: Transmission of tuberculosis (TB): TB is only transmitted by unvaccinated persons who reach the ${\mathcal {S}}$. The infection transmission coefficient or TB incidence rate is represented by $\beta$. This value represents the rate at which susceptible people become infected with tuberculosis.
3. Infected Population: The infected population is split into two groups: DS and MDR TB. These compartments are indicated by the symbols ${\mathcal {I}}_{s}$ and ${\mathcal {I}}_{r}$. DS-TB and MDR-TB are two types of tuberculosis with differing drug resistance profiles.
4. Drug Resistance: A subset of DS-TB patients transmission to the MDR-TB compartment ${\mathcal {I}}_{r}$. In a fraction of TB patients, this change signals the development of medication resistance.
5. Isolated Class: ${\mathcal {Q}}$ is a novel compartment established to reflect ${\mathcal {I}}_{r}$ patients that require specialized diagnosis and therapy. ${\mathcal {I}}_{r}$ patients are anticipated to be isolated from the general population at a rate given by. This isolation is an important step in limiting the spread of ${\mathcal {I}}_{r}$.
6. Recovery Class: Recovery rates for ${\mathcal {I}}_{s}$ and ${\mathcal {I}}_{r}$, and the isolated class ${\mathcal {Q}}$ are defined differently. These parameters describe the rates at which people recover from tuberculosis and progress to the recovered class ${\mathcal {R}}$. It is important to note that some healed people re-enter the susceptible class ${\mathcal {S}}$, since individuals may not gain full immunity following recovery.
7. To summarise, the model seeks to represent the dynamics of tuberculosis transmission, drug resistance development, isolation of ${\mathcal {I}}_{r}$ patients, recovery, and death within the community. These components and characteristics can be used to assess the effectiveness of various interventions and strategies for TB control and elimination, with a particular emphasis on ${\mathcal {I}}_{r}$ in the context of India. The model may be further analysed and simulated to get insights into the dynamics of tuberculosis in the community as well as the efficacy of various control methods.

## Invariant region

To study invariant regions of model (1). By assuming total population by $W(t)={\mathcal {S}}^*(t)+ {{\mathcal {I}}_{s}}^*(t)+{{\mathcal {I}}_{r}}^*(t)+{\mathcal {Q}}^*(t)+{\mathcal {R}}^*(t).$ At *t* differentiate *W*(*t*), we get

4 $$\begin{aligned} \frac{dW(t)}{dt}=\frac{d{\mathcal {S}}^*}{dt}+\frac{d{{\mathcal {I}}_{s}}^*}{dt} +\frac{d{{\mathcal {I}}_{r}}^*}{dt}+\frac{d{\mathcal {Q}}^*}{dt}+\frac{d{\mathcal {R}}^*}{dt}. \end{aligned}$$

Putting the values of $\frac{d{\mathcal {S}}^*}{dt},\frac{d{{\mathcal {I}}_{s}}^*}{dt},\frac{d{{\mathcal {I}}_{r}}^*}{dt},\frac{d{\mathcal {Q}}^*}{dt}$ and $\frac{d{\mathcal {R}}^*}{dt}$ in the Eq. (4). Deduce to the following

5 $$\begin{aligned} \frac{dW(t)}{dt}=\mu +{\mathcal {A}}(1-\theta )W, \end{aligned}$$

the separation variable method applied to Eq. (5). We obtain

6 $$\begin{aligned} \frac{dW(t)}{{\mathcal {A}}(1-\theta )W}=dt. \end{aligned}$$

Then we have

7 $$\begin{aligned}{} & {} \int \frac{dW}{{\mathcal {A}}(1-\theta )W}\ge \int dt \end{aligned}$$

8 $$\begin{aligned}{} & {} \Rightarrow W(t)\ge e^Ce^{{\mathcal {A}}(1-\theta )} \Rightarrow W(t)\ge L e^{{\mathcal {A}}(1-\theta )}. \end{aligned}$$

where $L = e^C$. Applying $\lim _{t\rightarrow \infty } \, W(t)=\frac{\mu }{{\mathcal {A}}(1-\theta )}.$ This give $0\le W(t)\le \frac{\mu }{{\mathcal {A}}(1-\theta )}.$ The considered tuberculosis model is bounded in the below domain.

9 $$\begin{aligned} \Xi =\big \{({\mathcal {S}}^{*},{{\mathcal {I}}_{s}}^{*},{{\mathcal {I}}_{r}}^{*}, {\mathcal {Q}}^{*},{\mathcal {R}}^{*})\in {\mathcal {R}}_{+}^{5}: 0 \le W(t) \le \frac{\mu }{{\mathcal {A}}(1-\theta )}\big \}. \end{aligned}$$

## Positive solutions

To investigate the positivity of all variables of the model.

### Theorem 1

Suppose that ${\mathcal {S}}^{*}(0)\ge 0, {{\mathcal {I}}_{s}}^{*}(0)\ge 0,{{\mathcal {I}}_{r}}^{*}(0)\ge 0,{\mathcal {Q}}^{*}(0)\ge 0$, and ${\mathcal {R}}^{*}(0)\ge 0.$ At $t>0$ then we check the solutions of $({\mathcal {S}}^{*},{{\mathcal {I}}_{s}}^{*},{{\mathcal {I}}_{r}}^{*},{\mathcal {Q}}^{*},{\mathcal {R}}^{*})$, are also positive.

### Proof

Consider the first equation of (1). Then we have

10 $$\begin{aligned} \frac{d{\mathcal {S}}^{*}}{dt}= & {} (1-\theta ){\mathcal {A}}-\beta {{\mathcal {I}}_{s}} ^{*}{\mathcal {S}}^{*}-\beta {{\mathcal {I}}_{r}}^{*}{\mathcal {S}}^{*}-\mu {\mathcal {S}} ^{*}+\gamma {\mathcal {R}}^{*}\nonumber \\\Rightarrow & {} \frac{d{\mathcal {S}}^{*}}{dt}\ge -\mu {\mathcal {S}}^{*}\nonumber \\\Rightarrow & {} \frac{d{\mathcal {S}}^{*}}{(\mu ){\mathcal {S}}^{*}}\ge -dt. \end{aligned}$$

From the Eq. (10). We get

11 $$\begin{aligned} \Rightarrow {\mathcal {S}}^{*}(t)\ge e^Ce^{-(\theta +\zeta _{1}+\nu )t}. \end{aligned}$$

Then with some calculation, we get

12 $$\begin{aligned} \Rightarrow {\mathcal {S}}^{*}(t)\ge {\mathcal {S}}^{*}(0)e^{-(\mu ) t}. \end{aligned}$$

Which deduce to $\Rightarrow {\mathcal {S}}^{*}(t)\ge {\mathcal {S}}^{*}(0)e^{-(\mu )t}\ge 0.$ By the second equation Eq. (1).

13 $$\begin{aligned} \frac{d{{\mathcal {I}}_{s}}^{*}}{dt}= & {} \beta {{\mathcal {I}}_{s}}^{*}{\mathcal {S}}^{*}-(\mu +\theta _{1}+\xi +\alpha _{1}){{\mathcal {I}}_{s}}^{*}\nonumber \\\Rightarrow & {} \frac{d{{\mathcal {I}}_{s}}^{*}}{dt}\ge -(\mu +\theta _{1}+\xi +\alpha _{1}){{\mathcal {I}}_{s}}^{*}\nonumber \\\Rightarrow & {} \frac{d{{\mathcal {I}}_{s}}^{*}}{(\mu +\theta _{1}+\xi +\alpha _{1}){{\mathcal {I}}_{s}}^{*}}\ge -dt. \end{aligned}$$

Using separation variable method to the Eq. (13). Which deduce

14 $$\begin{aligned} \Rightarrow {{\mathcal {I}}_{s}}^{*}(t)\ge e^Ce^{-(\mu +\theta _{1}+\xi +\alpha _{1}) t}. \end{aligned}$$

By calculation, we obtained the following

15 $$\begin{aligned} \Rightarrow {{\mathcal {I}}_{s}}^{*}(t)\ge {{\mathcal {I}}_{s}}^{*}(0)e^{-(\mu +\theta _{1}+\xi +\alpha _{1})t}. \end{aligned}$$

Which shows to $\Rightarrow {{\mathcal {I}}_{s}}^{*}(t)\ge {{\mathcal {I}}_{s}}^{*}(0)e^{-(\mu +\theta _{1}+\xi +\alpha _{1})t}\ge 0.$ Assuming third equation of (2).

16 $$\begin{aligned} \frac{d{{\mathcal {I}}_{r}}^{*}}{dt}= & {} \beta {{\mathcal {I}}_{r}}^{*}{\mathcal {S}}^{*} -(\mu +\theta _{2}+\xi +\alpha _{2}){{\mathcal {I}}_{r}}^{*}+\xi {{\mathcal {I}}_{s}}^{*}\nonumber \\\Rightarrow & {} \frac{d{{\mathcal {I}}_{r}}^{*}}{dt}\ge -(\mu +\theta _{2}+\xi +\alpha _{2}){{\mathcal {I}}_{r}}^{*}\nonumber \\\Rightarrow & {} \frac{d{{\mathcal {I}}_{r}}^{*}}{(\mu +\theta _{2}+\xi +\alpha _{2}){{\mathcal {I}}_{r}}^{*}}\ge -dt. \end{aligned}$$

applying separation variable to Eq. (13). We get

17 $$\begin{aligned} \Rightarrow {{\mathcal {I}}_{r}}^{*}(t)\ge e^Ce^{-(\mu +\theta _{2}+\xi +\alpha _{2}) t}. \end{aligned}$$

By calculation, we obtained

18 $$\begin{aligned} \Rightarrow {{\mathcal {I}}_{r}}^{*}(t)\ge {{\mathcal {I}}_{r}}^{*}(0)e^{-(\mu +\theta _{2}+\xi +\alpha _{2})t}. \end{aligned}$$

which gives to $\Rightarrow {{\mathcal {I}}_{r}}^{*}(t)\ge {{\mathcal {I}}_{r}}^{*}(0)e^{-(\mu +\theta _{2}+\xi +\alpha _{2})t}\ge 0.$ With a similar process, we can get solutions for the equations 4th and 5th,

$$\begin{aligned}{} & {} {\mathcal {Q}}^{*}(t)\ge e^{-(\mu +\theta _{3}+\alpha _{3})}{\mathcal {Q}}^{*}(0)\ge 0,\\{} & {} {\mathcal {R}}^{*}(t)\ge e^{-(\mu +\gamma )}{\mathcal {R}}^{*}(0)\ge 0. \end{aligned}$$

which completes the proof. $\square$

## Disease-free equilibrium of the model

To study the disease-free equilibrium point of the model. By assuming

19 $$\begin{aligned} \frac{d{\mathcal {S}}^*}{dt}=\frac{d{{\mathcal {I}}_{s}}^{*}}{dt}=\frac{d{{\mathcal {I}}_{r}}^{*}}{dt} =\frac{d{\mathcal {Q}}^*}{dt}=\frac{d{\mathcal {R}}^*}{dt}=0. \end{aligned}$$

Now, considering $\mathcal {Z}_{0}$ is free equilibrium point for (2). Such that

20 $$\begin{aligned} \mathcal {Z}_{0}({\mathcal {S}},{\mathcal {I}}_{r},{\mathcal {I}}_{s},{\mathcal {Q}},{\mathcal {R}}) =({\mathcal {S}}_{0},{{\mathcal {I}}_{r}}_{0},{{\mathcal {I}}_{s}}_{0},{\mathcal {I}}_{0},{\mathcal {R}}_{0}). \end{aligned}$$

here we considered for the simplicity in Eq. (2),

$$\begin{aligned} \hbar _{1}= & {} \mu +\theta _{1}+\xi +\alpha _{1},\\ \hbar _{2}= & {} \mu +\theta _{2}+\xi +\alpha _{2},\\ \hbar _{3}= & {} \mu +\theta _{3}+\alpha _{3}. \end{aligned}$$

By simplification and calculation, we obtained the below values

$$\begin{aligned} {\mathcal {S}}_{0}= & {} \frac{\hbar _{1}}{\beta },\\ {\mathcal {Q}}_{0}= & {} \frac{\sigma }{\hbar _{3}}{{\mathcal {I}}_{r}}_{0},\\ {{\mathcal {I}}_{s}}_{0}= & {} \frac{(\hbar _{2}-\hbar _{1})}{\xi }{{\mathcal {I}}_{s}}_{0},\\ {\mathcal {R}}_{0}= & {} \frac{1}{\mu _{1}+\gamma }\left( \frac{\theta _{1}(\hbar _{2-\hbar _{1}})}{\xi }+\theta _{2}+\frac{\theta _{3}\sigma }{\hbar _{3}}\right) {{\mathcal {I}}_{r}}_{0},\\ {{\mathcal {I}}_{r}}_{0}= & {} \frac{{\mathcal {A}}(1-\theta )-\frac{\mu \hbar _{1}}{\beta }}{\frac{\hbar _{1}}{\xi }(\hbar _{2}-\hbar _{1})+\hbar _{1}-\frac{\gamma }{\mu +\gamma } {\frac{\theta _{1}(\hbar _{2}-\hbar _{1})}{\xi }}+\theta _{2}+\frac{\theta _{3}\sigma }{\hbar _{3}}}. \end{aligned}$$

Therefore, we get the disease free equilibrium point of the model (2),

21 $$\begin{aligned} \mathcal {Z}_{0}({\mathcal {S}},{\mathcal {I}}_{s},{\mathcal {I}}_{r},{\mathcal {Q}},{\mathcal {R}}) =({\mathcal {S}}_{0},{{\mathcal {I}}_{s}}_{0},{{\mathcal {I}}_{r}}_{0},{\mathcal {Q}},{\mathcal {R}}_{0}). \end{aligned}$$

## Measurement of reproduction numbers $R_{o}$

**Figure 3:**
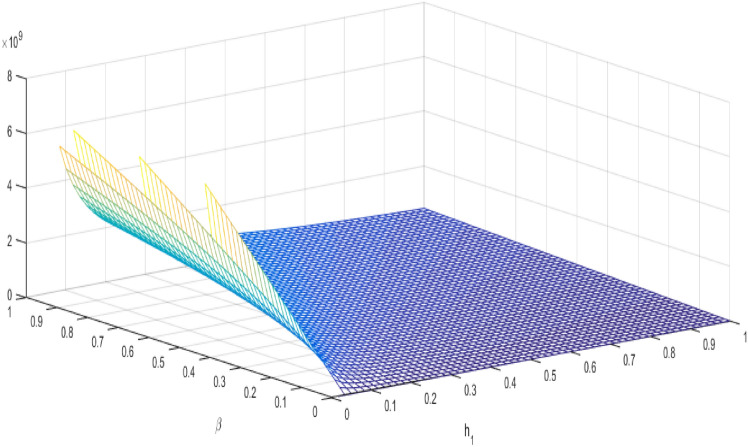
$R1_{0}$ ration of $\beta$ and $h_{1}$ of TB mathematical model.

**Figure 4:**
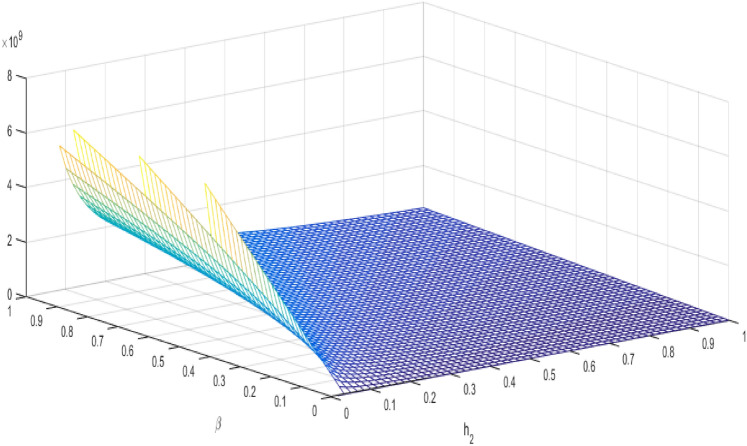
$R2_{0}$ ration of $\beta$ and $h_{2}$ of TB mathematical model.

This section focuses on the reproduction $R_{o}$ number for the model (2). We employ the next-generation matrix approach to find the reproduction number. We can see the dynamics of $R_{o}$ in Figs. 3 and 4. We define *F* and *V*, two matrices, from the model (2), Then we have

22 $$\begin{aligned} F= \begin{bmatrix} \beta \frac{{\mathcal {A}}(1-\theta )}{\mu } &{} \quad 0 &{} \quad 0\\ 0 &{} \quad \beta \frac{{\mathcal {A}}(1-\theta )}{\mu } &{} \quad 0\\ 0 &{} \quad 0 &{} \quad 0 \end{bmatrix}, V=\begin{bmatrix} \hbar _{1} &{} \quad 0 &{}\quad 0\\ -\xi &{} \quad \hbar _{2} &{} \quad 0\\ 0 &{} \quad -\sigma &{} \quad \hbar _{3} \end{bmatrix}. \end{aligned}$$

After calculation, we get the two reproduction numbers

### DS-TB

23 $$\begin{aligned} R1_{o}=\beta \frac{{\mathcal {A}}(1-\theta )}{\mu \hbar _{1}}. \end{aligned}$$

### MDR-TB

24 $$\begin{aligned} R2_{o}=\beta \frac{{\mathcal {A}}(1-\theta )}{\mu \hbar _{2}}. \end{aligned}$$

## Local stability of the model

This section focuses on studying the local stability of the considered model (2).

### Theorem 2

The point of diseases free equilibrium of the model (2) is said to be locally stable if and only if $R_{o}<1.$

### Proof

By using Jacobian matrix of model (2) to prove Theorem 2

25 $$\begin{aligned} \mathcal {J}_{E}= \begin{bmatrix} -(\beta {\mathcal {I}}_{s}+\beta {\mathcal {I}}_{r}+\mu ) &{} \quad -\beta {\mathcal {S}} &{} \quad -\beta {\mathcal {S}} &{} \quad 0 &{} \quad \gamma \\ \beta {\mathcal {I}}_{s} &{} \quad \beta {\mathcal {S}}-\hbar _{1} &{} \quad 0 &{} \quad 0 &{} \quad 0\\ \beta {\mathcal {I}}_{r} &{} \quad \xi &{} \quad \beta {\mathcal {S}}-\hbar _{2} &{} \quad 0 &{} \quad 0\\ 0 &{} \quad 0 &{} \quad \sigma &{} \quad -\hbar _{3} &{} \quad 0 \\ 0 &{} \quad \theta _{1} &{} \quad \theta _{2} &{} \quad \theta _{3} &{} \quad -(\mu +\gamma ) \\ \end{bmatrix}. \end{aligned}$$

Such that variational matrix for the diseases free equilibrium is given

26 $$\begin{aligned} \mathcal {J}_{E_{0}}= \begin{bmatrix} -\mu &{} \quad -\beta \frac{{\mathcal {A}}(1-\theta )}{\mu } &{} \quad -\beta \frac{{\mathcal {A}}(1-\theta )}{\mu } &{} \quad 0 &{} \quad \gamma \\ 0 &{} \quad \beta \frac{{\mathcal {A}}(1-\theta )}{\mu }-\hbar _{1} &{} \quad 0 &{} \quad 0 &{} \quad 0\\ 0 &{} \quad \xi &{} \quad \beta \frac{{\mathcal {A}}(1-\theta )}{\mu }-\hbar _{2} &{} \quad 0 &{} \quad 0\\ 0 &{} \quad 0 &{} \quad \sigma &{} \quad -\hbar _{3} &{} \quad 0 \\ 0 &{} \quad \theta _{1} &{} \quad \theta _{2} &{} \quad \theta _{3} &{} \quad -(\mu +\gamma ) \\ \end{bmatrix}. \end{aligned}$$

Eigenvalues for the matrix (27) for the diseases free equilibrium is given

27 $$\begin{aligned} \mathcal {J}_{E_{0}}= \begin{vmatrix} -\mu -\lambda _{1}&\quad -\beta \frac{{\mathcal {A}}(1-\theta )}{\mu }&\quad -\beta \frac{{\mathcal {A}} (1-\theta )}{\mu }&\quad 0&\quad \gamma \\ 0&\quad \beta \frac{{\mathcal {A}}(1-\theta )}{\mu }-\hbar _{1}-\lambda _{2}&\quad 0&\quad 0&\quad 0\\ 0&\quad \xi&\quad \beta \frac{{\mathcal {A}}(1-\theta )}{\mu }-\hbar _{2}-\lambda _{3}&\quad 0&\quad 0\\ 0&\quad 0&\quad \sigma&\quad -\hbar _{3}-\lambda _{4}&\quad 0 \\ 0&\quad \theta _{1}&\quad \theta _{2}&\quad \theta _{3}&\quad -(\mu +\gamma )-\lambda _{5} \\ \end{vmatrix}. \end{aligned}$$

The Eigenvalues of the matrix:

$$\begin{aligned} \lambda _{1}=-\mu , \lambda _{2}=\beta \frac{{\mathcal {A}}(1-\theta )}{\mu }-\hbar _{1}, \lambda _{3}=\beta \frac{{\mathcal {A}}(1-\theta )}{\mu }-\hbar _{2}, \lambda _{4}=-\hbar _{3}, \lambda _{5}=-(\mu +\gamma ). \end{aligned}$$

The values of $\lambda _{1}<0$, $\lambda _{4}<0$, and $\lambda _{5}<0$. But we do not know about $\lambda _{2}, \lambda _{3}$ to stable all the eigenvalues has to be less than 0. For this we have

28 $$\begin{aligned}{} & {} \beta \frac{{\mathcal {A}}(1-\theta )}{\mu }-\hbar _{1}<0\nonumber \\{} & {} \quad \Rightarrow \beta \frac{{\mathcal {A}}(1-\theta )}{\mu }<\hbar _{1},\nonumber \\{} & {} \quad \Rightarrow \beta \frac{{\mathcal {A}}(1-\theta )}{\mu \hbar _{1}}<1. \end{aligned}$$

Similarly for the $\lambda _{3},$ and we know its $R_{0}$ values, which shows that model (2), is locally asymptotically stable. $\square$

## Fractal fractional operators basic definitions

This section goes over some of the most recent fractal-fractional operator definitions. Some fundamental definitions of fractal fractional operators are used in this article, more information on fractal fractional operators can be found here^44–47^.

### Definition 1

^46^ Let $\mathcal {Y}(t)\in (a,b)$ a continuous function is given in the sense of $(0<\propto ,\sigma \le 1)$,Then fractal fractional Caputo and generalized Mittage-Leffler kernel

29 $$\begin{aligned}&_a^{ffm}\textbf{D}^{\propto ,\sigma }_{t}\mathcal {Y}(t)=\frac{{\textbf{N}}(\propto )}{[1-\propto ]}\int _a^{t}\frac{d\mathcal {Y}(\textrm{r})}{d\textrm{r}^{\sigma }} E_{\propto }\Big [-\frac{\propto }{1-\propto }(t-\textrm{r})^{\propto }\Big ]d\textrm{r},\\ {}&\text {where, }0<\propto ,\sigma \le 1,\,\,\,\, {\textbf{N}}(\propto ) =1-\propto +\propto /\Gamma (\propto ),\\ {}&\end{aligned}$$

generalized form is

30 $$\begin{aligned}&_a^{ffm}\textbf{D}^{\propto ,\sigma ,\theta }_{t}\digamma (t) =\frac{{\textbf{N}}(\propto )}{[1-\propto ]}\int _a^{t}\frac{d^{\theta } \digamma (\textrm{v})}{d\textrm{v}^{\sigma }}E_{\propto }\Big [-\frac{\propto }{1-\propto }(t-\textrm{v})^{\propto }\Big ]d\textrm{v},\,\,\,\,0<\theta \le 1.\\ \end{aligned}$$

where ${\textbf{N}}(\propto )$ is the normalization function which satisfies these properties, ${\textbf{N}}(0)={\textbf{N}}(1)=1.$

### Definition 2

^46^ The fractal fractional integral for any $\mathcal {Y}(t)\in (a,b)$ a continuous function is given in the sense of $(0<\propto ,\sigma \le 1)$, Mittage-Leffler kernel

31 $$\begin{aligned}&_a^{ffm}\textbf{J}^{\propto ,\sigma }_{0,t}\mathcal {Y}(t)=\frac{\sigma (1-\propto )t^{\sigma -1} \mathcal {Y}(t)}{{\textbf{N}}(\propto )}+\frac{\propto \sigma }{{\textbf{N}}(\propto )} \int _0^{t}(t-\textrm{r})^{\propto -1}\textrm{r}^{\propto -1}\mathcal {Y}(\textrm{r})d\textrm{r}. \end{aligned}$$

## Existence of solutions

The prominent of this article is to develop the existence of solutions and to illustrate the numerical outcomes of the fractal fractional order TB model. In the next section, our main objectives existence of results for the suggested model, and the next step to find unique solutions is our target for the TB model. For this, we define a Banach’s space. Consider $\mathcal {K}={\mathcal {I}}\times {\mathcal {R}}^5\rightarrow {\mathcal {R}}$, where $\mathbb {I}=[0,\tau ]$, as norm defined by $\Vert ({\mathcal {S}},{\mathcal {I}}_{s},{\mathcal {I}}_{r},{\mathcal {Q}},{\mathcal {R}})\Vert =\max _{t\in {\mathcal {I}}}\{|{\mathcal {S}}|+|{\mathcal {I}}_{s}|+|{\mathcal {I}}_{r}|+|{\mathcal {Q}}|+|{\mathcal {R}}|\}$ for $0<t<\tau <\infty$. Then, $(\mathcal {K},\Vert \Vert )$ clearly is a Banach’s space. By employing the fixed point theorem to study the existence of the solutions of the considered model. Therefore, considering the fractal fractional operator in place of the integer operator in the model (1), then we have

32 $$\begin{aligned} _{}^{ffm}\mathcal {D}^{\propto ,\sigma }_{0,t}({\mathcal {S}}(t))= & {} (1-\theta ){\mathcal {A}} -\beta {\mathcal {I}}_{s}(t){\mathcal {S}}(t)-\beta {\mathcal {I}}_{r}(t){\mathcal {S}}(t)-\mu {\mathcal {S}} (t)+\gamma {\mathcal {R}}(t),\nonumber \\ _{}^{ffm}\mathcal {D}^{\propto ,\sigma }_{0,t}({\mathcal {I}}_{s}(t))= & {} \beta {\mathcal {I}}_{s} (t){\mathcal {S}}(t)-\hbar _{1}{\mathcal {I}}_{s}(t),\nonumber \\ _{}^{ffm}\mathcal {D}^{\propto ,\sigma }_{0,t}({\mathcal {I}}_{r}(t))= & {} \beta {\mathcal {I}}_{r} (t){\mathcal {S}}(t)-\hbar _{2}{\mathcal {I}}_{r}(t)+\xi {\mathcal {I}}_{s}(t),\nonumber \\ _{}^{ffm}\mathcal {D}^{\propto ,\sigma }_{0,t}({\mathcal {Q}}(t))= & {} \sigma {\mathcal {I}}_{r}(t) -\hbar _{3}{\mathcal {Q}}(t)+\sigma {\mathcal {I}}_{r}(t),\nonumber \\ _{}^{ffm}\mathcal {D}^{\propto ,\sigma }_{0,t}({\mathcal {R}}(t))= & {} \theta _{1}{\mathcal {I}}_{s}(t) +\theta _{2}{\mathcal {I}}_{r}(t)+\theta _{3}{\mathcal {Q}}(t)-(\mu +\gamma ){\mathcal {R}}(t), \end{aligned}$$

with initial boundary value conditions

33 $$\begin{aligned}&{\mathcal {S}}(0)={\mathcal {S}}_{0},\,\,\,\,\,\,{\mathcal {I}}_{s}(0)={{\mathcal {I}}_{s}}_{0}, \,\,\,\,\,{\mathcal {I}}_{r}(0)={{\mathcal {I}}_{r}}_{0},\,\,\,\,\,\, {\mathcal {Q}}(0)={\mathcal {Q}}_{0},\,\,\,\,\,{\mathcal {R}}(0)={\mathcal {R}}_{0}, \end{aligned}$$

we construct the equations, like as

34 $$\begin{aligned}&\Theta _{1}(t,{\mathcal {S}}, {\mathcal {I}}_{s}, {\mathcal {I}}_{r}, {\mathcal {Q}}, {\mathcal {R}}) =(1-\theta ){\mathcal {A}}-\beta {\mathcal {I}}_{s}(t){\mathcal {S}}(t)-\beta {\mathcal {I}}_{r}(t) {\mathcal {S}}(t)-\mu {\mathcal {S}}(t)+\gamma {\mathcal {R}}(t),\\ {}&\Theta _{2}(t,{\mathcal {S}}, {\mathcal {I}}_{s}, {\mathcal {I}}_{r}, {\mathcal {Q}}, {\mathcal {R}}) =\beta {\mathcal {I}}_{s}(t){\mathcal {S}}(t)-\hbar _{1}{\mathcal {I}}_{s}(t),\\ {}&\Theta _{3}(z,{\mathcal {S}}, {\mathcal {I}}_{s}, {\mathcal {I}}_{r}, {\mathcal {Q}}, {\mathcal {R}}) =\beta {\mathcal {I}}_{r}(t){\mathcal {S}}(t)-\hbar _{2}{\mathcal {I}}_{r}(t)+\xi {\mathcal {I}}_{s}(t),\\ {}&\Theta _{4}(t,{\mathcal {S}}, {\mathcal {I}}_{s}, {\mathcal {I}}_{r}, {\mathcal {Q}}, {\mathcal {R}}) =\sigma {\mathcal {I}}_{r}(t)-\hbar _{3}{\mathcal {Q}}(t)+\sigma {\mathcal {I}}_{r}(t),\\ {}&\Theta _{5}(t,{\mathcal {S}}, {\mathcal {I}}_{s}, {\mathcal {I}}_{r}, {\mathcal {Q}}, {\mathcal {R}}) = \theta _{1}{\mathcal {I}}_{s}(t)+\theta _{2}{\mathcal {I}}_{r}(t)+\theta _{3}{\mathcal {Q}}(t) -(\mu +\gamma ){\mathcal {R}}(t),\\ {}&\end{aligned}$$

rearranging the above system (32), yields

35 $$\begin{aligned} _{}^{\mathcal {ABR}}\mathbb {D}_{0,t}(\zeta (t))= \chi (t,\zeta (t)). \end{aligned}$$

where $\zeta (t)$ and $\chi$ represent

36 $$\begin{aligned} \zeta (t)= \left\{ \begin{aligned}&{\mathcal {S}}(t)\\ {}&{\mathcal {I}}_{s}(t)\\ {}&{\mathcal {I}}_{r}(t) \\ {}&{\mathcal {Q}}(t)\\ {}&{\mathcal {R}}(t) \end{aligned}\right. \,\,\,\,\,\,\,and\,\,\,\,\, \chi \{t,\zeta (t)\}= \left\{ \begin{aligned}&\Theta _{1}(t,{\mathcal {S}}, {\mathcal {I}}_{s}, {\mathcal {I}}_{r}, {\mathcal {Q}}, {\mathcal {R}})\\ {}&\Theta _{2}(t,{\mathcal {S}}, {\mathcal {I}}_{s}, {\mathcal {I}}_{r}, {\mathcal {Q}}, {\mathcal {R}})\\ {}&\Theta _{3}(t,{\mathcal {S}}, {\mathcal {I}}_{s}, {\mathcal {I}}_{r}, {\mathcal {Q}}, {\mathcal {R}}) \\ {}&\Theta _{4}(t,{\mathcal {S}}, {\mathcal {I}}_{s}, {\mathcal {I}}_{r}, {\mathcal {Q}}, {\mathcal {R}})\\ {}&\Theta _{5}(t,{\mathcal {S}}, {\mathcal {I}}_{s}, {\mathcal {I}}_{r}, {\mathcal {Q}}, {\mathcal {R}}) \end{aligned}\right. \end{aligned}$$

employing Definition (1), to (35),

37 $$\begin{aligned}&\frac{{\textbf{N}}(\propto )}{1-\propto }\frac{d}{dt}\int _0^{t}\chi (\xi ,\zeta (\xi ))E_{\propto } \Big [-\frac{\propto }{1-\propto }(t-z)^{\propto }\Big ]dz=z t^{z-1}\chi (t,\zeta (t)) \end{aligned}$$

with the help of Definition (2), applying to (37), yields

38 $$\begin{aligned} \chi (t)=\chi (0)+\frac{1-\propto }{{\textbf{N}}(\propto )}z t^{z-1}\chi (t,\zeta (t)) +\frac{z\propto }{{\textbf{N}}(\propto )\Gamma (\propto )}\int _0^{t}(t-z)^{\propto -1} \chi (\xi ,\zeta (\xi ))\xi ^{z-1}dz. \end{aligned}$$

Consider here

39 $$\begin{aligned} \mathcal {B}_a^q=\mathcal {F}_{u}(t_{u})\times \overline{\mathcal {U}_{0}(\zeta _{0})}, \end{aligned}$$

where $\mathcal {F}_{u}=[t_{u-a},\,\,\,t_{u+a}]$ and $\overline{\mathcal {U}_{0}(\zeta _{0})}=[t_{0}-b,t_{0}+b]$. Now consider $\sup _{t\in \mathcal {B}_a^q}\Vert \chi \Vert =\mathcal {P}$,

By defining a norm

40 $$\begin{aligned} \Vert \xi \Vert _{\infty }=\sup _{t\in \mathcal {B}_a^q}|\xi (t)|. \end{aligned}$$

Consider one operator, $\mho :\mathcal {G}[\mathcal {F}_{u}(t_{u}), \mathcal {U}_{b}(t_{u})]\rightarrow \mathcal {G}[\mathcal {F}_{u}(b), \mathcal {U}_{b}(t_{u})]$, gives

41 $$\begin{aligned} \mho \zeta (t)=\zeta _0+\frac{1-\propto }{{\textbf{N}}(\propto )}z t^{z-1}\chi (t,\zeta (t)) +\frac{z\propto }{{\textbf{N}}(\propto )\Gamma (\propto )}\int _0^{t}(t-z)^{\propto -1}\chi (\xi ,\zeta (\xi ))\xi ^{z-1}dz, \end{aligned}$$

first, to show that $\Vert \mho \zeta (t)-\zeta _{0}\Vert <q$, such that

42 $$\begin{aligned} \Vert \mho \zeta (t)-\zeta _{0}\Vert\le & {} \frac{1-\propto }{{\textbf{N}}(\propto )}z t^{z-1}\Vert \chi (t,\zeta (t))\Vert \nonumber \\{} & {} +\frac{z\propto }{{\textbf{N}}(\propto )\Gamma (\propto )}\int _0^{t}(t-z)^{\propto -1}\Vert \chi (\xi ,\zeta (\xi )) \Vert \xi ^{z-1}dz\nonumber ,\\\le & {} \frac{1-\propto }{{\textbf{N}}(\propto )}z t^{z-1}\mathcal {P} +\frac{z\propto }{{\textbf{N}}(\propto )\Gamma (\propto )}\mathcal {P}\int _0^{t}(t-z)^{\propto -1}\xi ^{z-1}dz, \end{aligned}$$

suppose $\xi =tv$ and substitute in Eq. (42), we get

$$\begin{aligned} \Vert \mho \zeta (t)-\zeta _{0}\Vert \le \frac{1-\propto }{{\textbf{N}}(\propto )}z t^{z-1}\mathcal {P} +\frac{z\propto \mathcal {P}}{{\textbf{N}}(\propto )\Gamma (\propto )}\xi ^{z+\propto -u}{\mathcal {Q}}(z,\propto ), \end{aligned}$$

deduce to,

$$\begin{aligned} \Vert \mho \zeta (t)-\zeta _{0}\Vert \le q \,\,\,\,\,\,\,\rightarrow \mathcal {P}<\frac{q{\mathcal {Q}}(z,\propto ){\textbf{N}}(\propto )\Gamma (\propto )}{(1-\propto )\Gamma (\alpha )z t^{z-1}+\propto zt^{z+\propto -u}}. \end{aligned}$$

Consider, $\zeta _{1},\zeta _{2}\in \mathcal {G}[\mathcal {H}_{n}(t_{n}),\mathcal {C}_{b}(t_{n})]$, then we get

$$\begin{aligned} \Vert \mho \zeta _{1}-\mho \zeta _{2}\Vert \le{} & {} \frac{1-\propto }{{\textbf{N}}(\propto )}z t^{z-1}\Vert \chi (t,\zeta _{1}(t))-\chi (t,\zeta _{2}(t))\Vert \\{} & {} +\frac{z\propto }{{\textbf{N}}(\propto )\Gamma (\propto )}\int _0^{t}(t-z) ^{\propto -1}\Vert \chi (t,\zeta _{1}(t))-\chi (t,\zeta _{2}(t))\Vert \xi ^{z-1}dz, \end{aligned}$$

as $\mho$ is a contraction, then we get

43 $$\begin{aligned} \Vert \mho \zeta _{1}-\mho \zeta _{2}\Vert \le{} & {} \frac{1-\propto }{{\textbf{N}}(\propto )}z t^{z-1}\mathcal {X} \Vert \zeta _{1}-\zeta _{2}\Vert _{\infty }\nonumber \\{} & {} +\frac{z\propto \mathcal {X}}{{\textbf{N}}(\propto )\Gamma (\propto )}\int _0^{t}(t-z)^{\propto -1} \Vert \zeta _{1}-\zeta _{2}\Vert _{\infty }\xi ^{z-1}dz.\nonumber \\ \le{} & {} \frac{1-\propto }{{\textbf{N}}(\propto )}z t^{z-1}\mathcal {X}\Vert \zeta _{1} -\zeta _{2}\Vert _{\infty }\nonumber \\{} & {} +\frac{z\propto \mathcal {X}}{{\textbf{N}}(\propto )\Gamma (\propto )}\Vert \zeta _{1} -\zeta _{2}\Vert _{\infty }t^{\propto +z-3}{\mathcal {Q}}(z,\propto ).\nonumber \\ \Vert \mho \zeta _{1}-\mho \zeta _{2}\Vert \le{} & {} \Big [\frac{1-\propto }{{\textbf{N}} (\propto )}z t^{z-1}\mathcal {X}+\frac{z\propto \mathcal {X}}{{\textbf{N}} (\propto )\Gamma (\propto )}t^{\propto +z-3}{\mathcal {Q}}(z,\propto )\Big ]\Vert \zeta _{1}-\zeta _{2}\Vert _{\infty }. \end{aligned}$$

Thus, $\mho$ is a contraction if

44 $$\begin{aligned} \Vert \mho \zeta _{1}-\mho \zeta _{2}\Vert _{\infty }\le \Vert \zeta _{1}-\zeta _{2}\Vert . \end{aligned}$$

Deduce to the following

45 $$\begin{aligned} \mathcal {X}<\frac{1}{\frac{1-\propto }{{\textbf{N}}(\propto )}zt^{z-1}+\frac{z\propto }{{\textbf{N}}(\propto )\Gamma (\propto )}t^{\propto +z-3}{\mathcal {Q}}(z,\propto )}, \end{aligned}$$

and

46 $$\begin{aligned} \mathcal {P}<\frac{1}{\frac{1-\propto }{{\textbf{N}}(\propto )}zt^{z-1}+\frac{z\propto }{{\textbf{N}}(\propto )\Gamma (\propto )}t^{\propto +z-3}{\mathcal {Q}}(z,\propto )}. \end{aligned}$$

This completes all the conditions of Unique solutions of the fractal fractional order TB system (32).

## Stability analysis

Further to investigate fractal fractional TB model (32), for the stability analysis, here we use a theoretical approach to Hyres-Ulam stability.

### Definition 3

Considered model (32), exhibits Hyres-Ulam stable. If for each $({\mathcal {S}}^{*},{{\mathcal {I}}_{s}}^{*},{{\mathcal {I}}_{s}}^{*},{\mathcal {Q}}^{*},{\mathcal {R}}^{*})$ $\exists$ $\hbar _{j}>0$,$j\in {\textbf{H}}^5_1$, such that we can identify a real number $\forall$ $\chi _{j}>0,\,\,\,j\in {\textbf{H}}^5_1$, satisfying the inequality below

47 $$\begin{aligned} {\left\{ \begin{array}{ll} |_{}^{ffm}\mathbb {D}^{\propto ,\sigma }_{t}{\mathcal {S}}^{*}(t)-\Theta _{1}(t,{\mathcal {S}}^{*})|\le \chi _{1},\\ |_{}^{ffm}\mathbb {D}^{\propto ,\sigma }_{t}{{\mathcal {I}}_{s}}^{*}(t)-\Theta _{2} (t,{{\mathcal {I}}_{s}}^{*})|\le \chi _{2},\\ |_{}^{ffm}\mathbb {D}^{\propto ,\sigma }_{t}{{\mathcal {I}}_{r}}^{*}(t)-\Theta _{3} (t,{{\mathcal {I}}_{r}}^{*})|\le \chi _{3},\\ |_{}^{ffm}\mathbb {D}^{\propto ,\sigma }_{t}{\mathcal {Q}}^{*}(t)-\Theta _{4}(t, {\mathcal {Q}}^{*})|\le \chi _{4},\\ |_{}^{ffm}\mathbb {D}^{\propto ,\sigma }_{t}{\mathcal {R}}^{*}(t)-\Theta _{5}(t, {\mathcal {R}}^{*})|\le \chi _{5}, \end{array}\right. } \end{aligned}$$

such that $\exists$ $({\mathcal {S}},{\mathcal {I}}_{s},{\mathcal {I}}_{s},{\mathcal {Q}},{\mathcal {R}})$ considered model (32), which gives

48 $$\begin{aligned} {\left\{ \begin{array}{ll} \Vert {\mathcal {S}}(t)-{\mathcal {S}}^{*}\Vert \le \hbar _{1}\chi _{1},\\ \Vert {\mathcal {I}}_{s}(t)-{{\mathcal {I}}_{s}}^{*}\Vert \le \hbar _{2}\chi _{2},\\ \Vert {\mathcal {I}}_{r}(t)-{{\mathcal {I}}_{r}}^{*}\Vert \le \hbar _{3}\chi _{3},\\ \Vert {\mathcal {Q}}(t)-{\mathcal {Q}}^{*}\Vert \le \hbar _{4}\chi _{4},\\ \Vert {\mathcal {R}}(t)-{\mathcal {R}}^{*}\Vert \le \hbar _{5}\chi _{5}, \end{array}\right. } \end{aligned}$$

where $\Theta _{j} \in {\textbf{H}}_{1}^{5}$, is mention in (34).

### Remark 1

Let (47) is a solution ${\mathcal {S}}^{*}$ iff $\exists$ $\Pi _{1}$ such that, if it justify condition outlined below

49 $$\begin{aligned}&(1)\;  |\Pi _{1}(t)|<\chi _{1},\nonumber \\&(2) \; ^{ffm}\mathbb {D}^{\propto ,\sigma }_{t}{\mathcal {S}}^{*}(t) =\Theta _{1}(t,{\mathcal {S}}^{*})|\le \chi _{1}+ \Pi _{1}(t). \end{aligned}$$

### Theorem 3

Let the model (32) be Hyres-Ulam stable if the below inequality satisfy

50 $$\begin{aligned} \lambda _{i}[\frac{\propto \sigma \Gamma (\sigma )+\sigma (1-\propto )\Gamma (\propto +\sigma ) }{{\textbf{N}}(\propto )\Gamma (\propto +\sigma )}]\le 1, i\in {\textbf{H}}_1^{5}. \end{aligned}$$

### Proof

let ${\mathcal {S}}^{*}$, and $\chi >0$. Then the following inequality holds have

51 $$\begin{aligned} |_{}^{ffm}\mathbb {D}^{\propto ,\sigma }_{t}{\mathcal {S}}^{*}(t)-\Theta _{1}(t,{\mathcal {S}}^{*})|\le \chi _{1}. \end{aligned}$$

with the help of Remark (5.2), the (10) gives the below form

52 $$\begin{aligned} {}^{ffm}\mathbb {D}^{\propto ,\sigma }_{t}{\mathcal {S}}^{*}(t)=\Theta _{1}(t,{\mathcal {S}}^{*})|\le \chi _{1}+ \Pi _{1}(t). \end{aligned}$$

Utilizing integral (2), to Eq. (52). Then we get

53 $$\begin{aligned} {\textbf{S}}^{*}(t)= & {} {\textbf{S}}^{0} +\frac{\propto \sigma }{{\textbf{N}}(\propto )\Gamma (\propto )}\int _0^{t}(t-\xi ) ^{\propto -1}\xi ^{\sigma -1}\Theta _{1}(\xi ,{\mathcal {S}}^{*}(\xi ))d\xi + \frac{(1-\propto )\sigma }{{\textbf{N}}(\propto )}t^{\sigma -1}\Theta _{1}(\xi ,{\mathcal {S}}^{*}(t))\nonumber \\{} & {} +\frac{\propto \sigma }{{\textbf{N}}(\propto )\Gamma (\propto )}\int _0^{t}(t-\xi ) ^{\propto -1}\xi ^{\sigma -1}\Pi _{1}(\xi )d\xi +\frac{(1-\propto )\sigma }{{\textbf{N}}(\propto )}t^{\sigma -1}\Pi _{1}(t). \end{aligned}$$

Let ${\textbf{S}}$ be a unique solution, for the considered model (32). We obtain

54 $$\begin{aligned} {\mathcal {S}}(t)={\textbf{S}}^{0} +\frac{\propto \sigma }{{\textbf{N}}(\propto )\Gamma (\propto )}\int _0^{t}(t-\xi ) ^{\propto -1}\xi ^{\sigma -1}\Theta _{1}(\xi ,{\textbf{S}}(\xi ))d\xi +\frac{(1-\propto ) \sigma }{{\textbf{N}}(\propto )}t^{\sigma -1}\Theta _{1}(\xi ,{\textbf{S}}(t)). \end{aligned}$$

Therefore,

55 $$\begin{aligned} |{\mathcal {S}}^{*}(t)-{\mathcal {S}}(t)|\le & {} \frac{\propto \sigma }{{\textbf{N}}(\propto ) \Gamma (\propto )}\int _0^{t}(t-\xi )^{\propto -1}\xi ^{\sigma -1}|\Theta _{1}(\xi , {\mathcal {S}}^{*}(\xi ))-\Theta _{1}(\xi ,{\mathcal {S}}(\xi ))|d\xi \nonumber \\{} & {} +\frac{(1-\propto )\sigma }{{\textbf{N}}(\propto )}t^{\sigma -1}|\Theta _{1}(\xi , {\mathcal {S}}^{*}(t))-Theta_{1}(\xi ,{\mathcal {S}}(t))| \end{aligned}$$

56 $$\begin{aligned}+ & {} \frac{\propto \sigma }{{\textbf{N}}(\propto )\Gamma (\propto )}\int _0^{t}(t-\xi ) ^{\propto -1}\xi ^{\sigma -1}|\Pi _{1}(\xi )|d\xi \nonumber \\+ & {} \frac{(1-\propto )\sigma }{{\textbf{N}}(\propto )}t^{\sigma -1}|\Pi _{1}(t)|,\nonumber \\\le & {} \lambda _{i}[\frac{\propto \sigma \Gamma (\sigma )+\sigma (1-\propto )\Gamma (\propto +\sigma ) }{{\textbf{N}}(\propto )\Gamma (\propto +\sigma )}]|{\textbf{S}}^{*}(t)-{\textbf{S}}(t)|\nonumber \\{} & {} +[\frac{\propto \sigma \Gamma (\sigma )+\sigma (1-\propto )\Gamma (\propto +\sigma ) }{{\textbf{N}}(\propto )\Gamma (\propto +\sigma )}]\chi _{1}. \end{aligned}$$

Further,

57 $$\begin{aligned} \Vert {\mathcal {S}}^{*}(t)-{\mathcal {S}}(t)\Vert\le & {} \frac{ \left[ \frac{\propto \sigma \Gamma (\sigma ) +\sigma (1-\propto )\Gamma (\propto +\sigma ) }{{\textbf{N}}(\propto )\Gamma (\propto +\sigma )} \right] \chi _{1}}{1- \left[ \frac{\propto \sigma \Gamma (\sigma )+\sigma (1-\propto )\Gamma (\propto +\sigma ) }{{\textbf{N}}(\propto )\Gamma (\propto +\sigma )} \right] \lambda _{1}}. \end{aligned}$$

Let consider

58 $$\begin{aligned} \hbar _{1}\le & {} \frac{ \left[ \frac{\propto \sigma \Gamma (\sigma )+\sigma (1-\propto )\Gamma (\propto +\sigma ) }{{\textbf{N}}(\propto )\Gamma (\propto +\sigma )} \right] }{1- \left[ \frac{\propto \sigma \Gamma (\sigma )+\sigma (1-\propto ) \Gamma (\propto +\sigma ) }{{\textbf{N}}(\propto )\Gamma (\propto +\sigma )} \right] \lambda _{1}}. \end{aligned}$$

This leads $\Vert {\mathcal {S}}^{*}-{\mathcal {S}}\Vert \le \lambda _{1}\chi _{1}.$ Similarly

$$\begin{aligned} \Vert {{\mathcal {I}}_{s}}^{*}-{\mathcal {I}}_{s}\Vert \le \lambda _{2}\chi _{2},\,\,\,\,\,\,\,\,\Vert {{\mathcal {I}}_{r}}^{*}-{\mathcal {I}}_{r}\Vert \le \lambda _{3}\chi _{3}\\ \Vert {\mathcal {Q}}^{*}-{\mathcal {Q}}\Vert \le \lambda _{4}\chi _{4},\,\,\,\,\,\,\,\,\Vert {\mathcal {R}} ^{*}-{\mathcal {R}}\Vert \le \lambda _{5}\chi _{5}. \end{aligned}$$

Finally, the conditions are satisfied therefore, the system (32) is Hyres-Ulam stable. $\square$

## Iterative numerical Scheme

By considering the system (32), in the sense of fractal fractional operator

59 $$\begin{aligned} _{}^{ffm}\mathbb {D}^{\propto ,\sigma }_{0,t}({\mathcal {S}}(t))= & {} (1-\theta ) {\mathcal {A}}-\beta {\mathcal {I}}_{s}(t){\mathcal {S}}(t)-\beta {\mathcal {I}}_{r}(t) {\mathcal {S}}(t)-\mu {\mathcal {S}}(t)+\gamma {\mathcal {R}}(t),\nonumber \\ _{}^{ffm}\mathbb {D}^{\propto ,\sigma }_{0,t}({\mathcal {I}}_{s}(t))= & {} \beta {\mathcal {I}}_{s} (t){\mathcal {S}}(t)-(\mu +\theta _{1}+\xi +\alpha _{1}){\mathcal {I}}_{s}(t),\nonumber \\ _{}^{ffm}\mathbb {D}^{\propto ,\sigma }_{0,t}({\mathcal {I}}_{r}(t))= & {} \beta {\mathcal {I}}_{r} (t){\mathcal {S}}(t)-(\mu +\theta _{2}+\xi +\alpha _{2}){\mathcal {I}}_{r}(t)+\xi {\mathcal {I}}_{s}(t),\nonumber \\ _{}^{ffm}\mathbb {D}^{\propto ,\sigma }_{0,t}({\mathcal {Q}}(t))= & {} \sigma {\mathcal {I}}_{r}(t) -(\mu +\theta _{3}+\alpha _{3}){\mathcal {Q}}(t)+\sigma {\mathcal {I}}_{r}(t),\nonumber \\ _{}^{ffm}\mathbb {D}^{\propto ,\sigma }_{0,t}({\mathcal {R}}(t))= & {} \theta _{1}{\mathcal {I}}_{s}(t) +\theta _{2}{\mathcal {I}}_{r}(t)+\theta _{3}{\mathcal {Q}}(t)-(\mu +\gamma ){\mathcal {R}}(t). \end{aligned}$$

For the simplicity, we consider

$$\begin{aligned}&\Theta _{1}(z,{\mathcal {S}}, {\mathcal {I}}_{s}, {\mathcal {I}}_{r}, {\mathcal {Q}}, {\mathcal {R}}) =(1-\theta ){\mathcal {A}}-\beta {\mathcal {I}}_{s}(t){\mathcal {S}}(t)-\beta {\mathcal {I}}_{r}(t) {\mathcal {S}}(t)-\mu {\mathcal {S}}(t)+\gamma {\mathcal {R}}(t),\nonumber \\ {}&\Theta _{2}(z,{\mathcal {S}}, {\mathcal {I}}_{s}, {\mathcal {I}}_{r}, {\mathcal {Q}}, {\mathcal {R}}) =\beta {\mathcal {I}}_{s}(t){\mathcal {S}}(t)-(\mu +\theta _{1}+\xi +\alpha _{1}){\mathcal {I}}_{s}(t),\\ {}&\Theta _{3}(z,{\mathcal {S}}, {\mathcal {I}}_{s}, {\mathcal {I}}_{r}, {\mathcal {Q}}, {\mathcal {R}}) =\beta {\mathcal {I}}_{r}(t){\mathcal {S}}(t)-(\mu +\theta _{2}+\xi +\alpha _{2}){\mathcal {I}}_{r} (t)+\xi {\mathcal {I}}_{s}(t),\\ {}&\Theta _{4}(z,{\mathcal {S}}, {\mathcal {I}}_{s}, {\mathcal {I}}_{r}, {\mathcal {Q}}, {\mathcal {R}}) =\sigma {\mathcal {I}}_{r}(t)-(\mu +\theta _{3}+\alpha _{3}){\mathcal {Q}}(t)+\sigma {\mathcal {I}}_{r}(t),\\ {}&\Theta _{5}(z,{\mathcal {S}}, {\mathcal {I}}_{s}, {\mathcal {I}}_{r}, {\mathcal {Q}}, {\mathcal {R}}) = \theta _{1}{\mathcal {I}}_{s}(t)+\theta _{2}{\mathcal {I}}_{r}(t)+\theta _{3}{\mathcal {Q}}(t)-(\mu +\gamma ) {\mathcal {R}}(t), \end{aligned}$$

employing the $\mathcal{A}\mathcal{B}$-integral to Eq. (59), which yields

60 $$\begin{aligned} {\mathcal {S}}(t)-{\mathcal {S}}(0)= & {} \frac{\sigma t^{\sigma -1}(1-\propto )\Theta _{1} (t,{\mathcal {S}}, {\mathcal {I}}_{s}, {\mathcal {I}}_{r}, {\mathcal {Q}}, {\mathcal {R}})}{{\textbf{N}}(\propto )}\nonumber \\{} & {} + \, \frac{\propto \sigma }{{\textbf{N}}(\propto )\Gamma (\propto )}\int _0^{t}(t-z)^{\propto -1}\Theta _{1} (z,{\mathcal {S}}, {\mathcal {I}}_{s}, {\mathcal {I}}_{r}, {\mathcal {Q}}, {\mathcal {R}})z^{\sigma -1}dz,\nonumber \\ {\mathcal {I}}_{s}(t)-{\mathcal {I}}_{s}(0)= & {} \frac{\sigma t^{\sigma -1}(1-\propto )\Theta _{2}(z,{\mathcal {S}}, {\mathcal {I}}_{s}, {\mathcal {I}}_{r}, {\mathcal {Q}}, {\mathcal {R}})}{{\textbf{N}}(\propto )}\nonumber \\{} & {} + \, \frac{\propto \sigma }{{\textbf{N}}(\propto )\Gamma (\propto )}\int _0^{t}(t-z)^{\propto -1}\Theta _{2}(z, {\mathcal {S}}, {\mathcal {I}}_{s}, {\mathcal {I}}_{r}, {\mathcal {Q}}, {\mathcal {R}})z^{\sigma -1}dz,\nonumber \\ {\mathcal {I}}_{r}(t)-{\mathcal {I}}_{r}(0)= & {} \frac{\sigma t^{\sigma -1}(1-\propto )\Theta _{3}(t,{\mathcal {S}}, {\mathcal {I}}_{s}, {\mathcal {I}}_{r}, {\mathcal {Q}}, {\mathcal {R}})}{{\textbf{N}}(\propto )}\nonumber \\{} & {} + \, \frac{\propto \sigma }{{\textbf{N}}(\propto )\Gamma (\propto )}\int _0^{t}(t-z)^{\propto -1}\Theta _{3} (z,{\mathcal {S}}, {\mathcal {I}}_{s}, {\mathcal {I}}_{r}, {\mathcal {Q}}, {\mathcal {R}})z^{\sigma -1}dz,\nonumber \\ {\mathcal {Q}}(t)-{\mathcal {Q}}(0)= & {} \frac{\sigma t^{\sigma -1}(1-\propto )\Theta _{4}(z,{\mathcal {S}}, {\mathcal {I}}_{s}, {\mathcal {I}}_{r}, {\mathcal {Q}}, {\mathcal {R}})}{{\textbf{N}}(\propto )}\nonumber \\{} & {} + \, \frac{\propto \sigma }{{\textbf{N}}(\propto )\Gamma (\propto )}\int _0^{t}(t-z)^{\propto -1}\Theta _{4} (z,{\mathcal {S}}, {\mathcal {I}}_{s}, {\mathcal {I}}_{r}, {\mathcal {Q}}, {\mathcal {R}})z^{\sigma -1}dz,\nonumber \\ {\mathcal {R}}(t)-{\mathcal {R}}(0)= & {} \frac{\sigma t^{\sigma -1}(1-\propto )\Theta _{5}(t,{\mathcal {S}}, {\mathcal {I}}_{s}, {\mathcal {I}}_{r}, {\mathcal {Q}}, {\mathcal {R}})}{{\textbf{N}}(\propto )}\nonumber \\{} & {} + \, \frac{\propto \sigma }{{\textbf{N}}(\propto )\Gamma (\propto )}\int _0^{t}(t-z)^{\propto -1}\Theta _{5} (z,{\mathcal {S}}, {\mathcal {I}}_{s}, {\mathcal {I}}_{r}, {\mathcal {Q}}, {\mathcal {R}})z^{\sigma -1}dz. \end{aligned}$$

By data fitting $t=t_{n+1}$ in Eq. (60), we obtain the below form

61 $$\begin{aligned} {\mathcal {S}}^{n+1}(t)= & {} {\mathcal {S}}^{0}+\frac{\sigma t_{n}^{\sigma -1}(1-\propto )\Theta _{1}(t_{n}, {\mathcal {S}}^{n}, {{\mathcal {I}}_{s}}^{n}, {{\mathcal {I}}_{r}}^{n}, {\mathcal {Q}}^{n}, {\mathcal {R}}^{n})}{{\textbf{N}}(\propto )}\nonumber \\{} & {} +\frac{\propto \sigma }{{\textbf{N}}(\propto )\Gamma (\propto )}\int _0^{t_{n+1}}(t_{n+1}-z)^{\propto -1} \Theta _{1}(z,{\mathcal {S}}, {\mathcal {I}}_{s}, {\mathcal {I}}_{r}, {\mathcal {Q}}, {\mathcal {R}})z^{\sigma -1}dz,\nonumber \\ {{\mathcal {I}}_{s}}^{n+1}(t)= & {} {{\mathcal {I}}_{s}}^{0}+\frac{\sigma t_{n}^{\sigma -1}(1-\propto )\Theta _{2} (t_{n},{\mathcal {S}}^{n}, {{\mathcal {I}}_{s}}^{n}, {{\mathcal {I}}_{r}}^{n}, {\mathcal {Q}}^{n}, {\mathcal {R}}^{n})}{{\textbf{N}}(\propto )}\nonumber \\{} & {} +\frac{\propto \sigma }{{\textbf{N}}(\propto )\Gamma (\propto )}\int _0^{t_{n+1}}(t_{n+1}-z)^{\propto -1} \Theta _{2}(z,{\mathcal {S}}, {\mathcal {I}}_{s}, {\mathcal {I}}_{r}, {\mathcal {Q}}, {\mathcal {R}}) z^{\sigma -1}dz,\nonumber \\ {{\mathcal {I}}_{r}}^{n+1}(t)= & {} {{\mathcal {I}}_{r}}^{0}+\frac{\sigma t_{n}^{\sigma -1}(1-\propto )\Theta _{3} (t_{n},{\mathcal {S}}^{n}, {{\mathcal {I}}_{s}}^{n}, {{\mathcal {I}}_{r}}^{n}, {\mathcal {Q}}^{n}, {\mathcal {R}}^{n})}{{\textbf{N}}(\propto )}\nonumber \\{} & {} +\frac{\propto \sigma }{{\textbf{N}}(\propto )\Gamma (\propto )}\int _0^{t_{n+1}}(t_{n+1}-z)^{\propto -1} \Theta _{3}(z,{\mathcal {S}}, {\mathcal {I}}_{s}, {\mathcal {I}}_{r}, {\mathcal {Q}}, {\mathcal {R}}) z^{\sigma 
-1}dz,\nonumber \\ {\mathcal {Q}}^{n+1}(t)= & {} {\mathcal {Q}}^{0}+\frac{\sigma t_{n}^{\sigma -1}(1-\propto )\Theta _{4}(t_{n}, {\mathcal {S}}^{n}, {{\mathcal {I}}_{s}}^{n}, {{\mathcal {I}}_{r}}^{n}, {\mathcal {Q}}^{n}, {\mathcal {R}}^{n})}{{\textbf{N}}(\propto )}\nonumber \\{} & {} +\frac{\propto \sigma }{{\textbf{N}}(\propto )\Gamma (\propto )}\int _0^{t_{n+1}}(t_{n+1}-z) ^{\propto -1}\Theta _{4}(z,{\mathcal {S}}, {\mathcal {I}}_{s}, {\mathcal {I}}_{r}, {\mathcal {Q}}, {\mathcal {R}})z^{\sigma -1}dz,\nonumber \\ {\mathcal {R}}^{n+1}(t)= & {} {\mathcal {R}}^{0}+\frac{\sigma t^{\sigma -1}(1-\propto )\Theta _{5}(t_{n}, {\mathcal {S}}^{n}, {{\mathcal {I}}_{s}}^{n}, {{\mathcal {I}}_{r}}^{n}, {\mathcal {Q}}^{n}, {\mathcal {R}}^{n})}{{\textbf{N}}(\propto )}\nonumber \\{} & {} +\frac{\propto \sigma }{{\textbf{N}}(\propto )\Gamma (\propto )}\int _0^{t_{n+1}}(t_{n+1}-z) ^{\propto -1}\Theta _{5}(z,{\mathcal {S}}, {\mathcal {I}}_{s}, {\mathcal {I}}_{r}, {\mathcal {Q}}, {\mathcal {R}})z^{\sigma -1}dz. \end{aligned}$$

In (61), by approximating the integral, which give the below

62 $$\begin{aligned} {\mathcal {S}}^{n+1}(t)= & {} {\mathcal {S}}^{0}+\frac{\sigma t_{n}^{\sigma -1}(1-\propto ) \Theta _{1}(t_{n},{\mathcal {S}}^{n}, {{\mathcal {I}}_{s}}^{n}, {{\mathcal {I}}_{r}}^{n}, {\mathcal {Q}}^{n}, {\mathcal {R}}^{n})}{{\textbf{N}}(\propto )}\nonumber \\{} & {} + \, \frac{\propto \sigma }{{\textbf{N}}(\propto )\Gamma (\propto )}\sum _{r=0}^{q}\int _{t_{r}} ^{t_{r+1}}(t_{n+1}-z)^{\propto -1}\Theta _{1}(z,{\mathcal {S}}, {\mathcal {I}}_{s}, {\mathcal {I}}_{r}, {\mathcal {Q}}, {\mathcal {R}})z^{\sigma -1}dz,\nonumber \\ {{\mathcal {I}}_{s}}^{n+1}(t)= & {} {{\mathcal {I}}_{s}}^{0}+\frac{\sigma t_{n}^{\sigma -1}(1-\propto ) \Theta _{2}(t_{n},{\mathcal {S}}^{n}, {{\mathcal {I}}_{s}}^{n}, {{\mathcal {I}}_{r}}^{n}, {\mathcal {Q}}^{n}, {\mathcal {R}}^{n})}{{\textbf{N}}(\propto )}\nonumber \\{} & {} + \, \frac{\propto \sigma }{{\textbf{N}}(\propto )\Gamma (\propto )}\sum _{r=0}^{q}\int _{t_{r}}^{t_{r+1}} (t_{n+1}-z)^{\propto -1}\Theta _{2}(z,{\mathcal {S}}, {\mathcal {I}}_{s}, {\mathcal {I}}_{r}, {\mathcal {Q}}, {\mathcal {R}})z^{\sigma -1}dz,\nonumber \\ {{\mathcal {I}}_{r}}^{n+1}(t)= & {} {{\mathcal {I}}_{r}}^{0}+\frac{\sigma t_{n}^{\sigma -1}(1-\propto ) \Theta _{3}(t_{n},{\mathcal {S}}^{n}, {{\mathcal {I}}_{s}}^{n}, {{\mathcal {I}}_{r}}^{n}, {\mathcal {Q}}^{n}, {\mathcal {R}}^{n})}{{\textbf{N}}(\propto )}\nonumber \\{} & {} + \, \frac{\propto \sigma }{{\textbf{N}}(\propto )\Gamma (\propto )}\sum _{r=0}^{q}\int _{t_{r}}^{t_{r+1}} (t_{n+1}-z)^{\propto -1}\Theta _{3}(z,{\mathcal {S}}, {\mathcal {I}}_{s}, {\mathcal {I}}_{r}, {\mathcal {Q}}, {\mathcal {R}})z^{\sigma -1}dz,\nonumber \\ {\mathcal {Q}}^{n+1}(t)= & {} {\mathcal {Q}}^{0}+\frac{\sigma t_{n}^{\sigma -1}(1-\propto )\Theta _{4}(t_{n}, {\mathcal {S}}^{n}, {{\mathcal {I}}_{s}}^{n}, {{\mathcal {I}}_{r}}^{n}, {\mathcal {Q}}^{n}, 
{\mathcal {R}}^{n})}{{\textbf{N}}(\propto )}\nonumber \\{} & {} + \, \frac{\propto \sigma }{{\textbf{N}}(\propto )\Gamma (\propto )}\sum _{r=0}^{q}\int _{t_{r}}^{t_{r+1}} (t_{n+1}-z)^{\propto -1}\Theta _{4}(z,{\mathcal {S}}, {\mathcal {I}}_{s}, {\mathcal {I}}_{r}, {\mathcal {Q}}, {\mathcal {R}})z^{\sigma -1}dz,\nonumber \\ {\mathcal {R}}^{n+1}(t)= & {} {\mathcal {R}}^{0}+\frac{\sigma t^{\sigma -1}(1-\propto )\Theta _{5}(t_{n}, {\mathcal {S}}^{n}, {{\mathcal {I}}_{s}}^{n}, {{\mathcal {I}}_{r}}^{n}, \mathcal {Q
}^{n}, {\mathcal {R}}^{n})}{{\textbf{N}}(\propto )}\nonumber \\{} & {} + \, \frac{\propto \sigma }{{\textbf{N}}(\propto )\Gamma (\propto )}\sum _{r=0}^{q}\int _{t_{r}}^{t_{r+1}} (t_{n+1}-z)^{\propto -1}\Theta _{5}(z,{\mathcal {S}}, {\mathcal {I}}_{s}, {\mathcal {I}}_{r}, {\mathcal {Q}}, {\mathcal {R}})z^{\sigma -1}dz. \end{aligned}$$

By utilizing Lagrangian interpolation polynomial to discretiz, we get below form

63 $$\begin{aligned} {\mathcal {S}}^{n+1}(t)= & {} \, {\mathcal {S}}^{0}+\frac{\sigma t_{n}^{\sigma -1}(1-\propto )\Theta _{1} (t_{n},{\mathcal {S}}^{n}, {{\mathcal {I}}_{s}}^{n}, {{\mathcal {I}}_{r}}^{n}, {\mathcal {Q}}^{n}, {\mathcal {R}}^{n})}{{\textbf{N}}(\propto )}\nonumber \\{} & {} +  \, \frac{\sigma ({\textbf{Q}} t)^{\propto }}{{\textbf{N}}(\propto )\Gamma (\propto +2)}\sum _{\varrho =0}^{q} \Big [t_{\varrho }^{\sigma -1}\Theta _{1}(t_{\varrho },{\mathcal {S}}^{\varrho }, {{\mathcal {I}}_{s}}^{\varrho }, {{\mathcal {I}}_{r}}^{\varrho }, {\mathcal {Q}}^{\varrho }, {\mathcal {R}}^{\varrho })\nonumber \\{} & {} \times \, ((q+1-\varrho )^{\propto }(q-\varrho +2+\propto ) -(q-\varrho )^{\propto }(p-\varrho +2+2\propto ))\nonumber \\{} & {} - \, t_{\varrho -1}^{\sigma -1} \Theta _{1}(t_{\varrho },{\mathcal {S}}^{\varrho -1}, {{\mathcal {I}}_{s}}^{\varrho -1}, {{\mathcal {I}}_{r}}^{\varrho -1}, {\mathcal {Q}}^{\varrho -1}, {\mathcal {R}}^{\varrho -1})\nonumber \\{} & {} \times \, ((q-\varrho +1)^{\propto +1}-(p-\varrho )^{\propto }(p-\varrho +1+\propto )) \Big ],\nonumber \\ {{\mathcal {I}}_{s}}^{n+1}(t) =& \,{{\mathcal {I}}_{s}}^{0} +  {} \, \frac{\sigma t_{n}^{\sigma -1}(1-\propto ) \Theta _{2}(t_{n},{\mathcal {S}}^{n}, {{\mathcal {I}}_{s}}^{n}, {{\mathcal {I}}_{r}}^{n}, {\mathcal {Q}}^{n}, {\mathcal {R}}^{n})}{{\textbf{N}}(\propto )}\nonumber \\{} & {} + \, \frac{\sigma ({\textbf{Q}} t)^{\propto }}{{\textbf{N}}(\propto )\Gamma (\propto +2)}\sum _{\varrho =0}^{q} \Big [t_{\varrho }^{\sigma -1}\Theta _{2}(t_{\varrho },{\mathcal {S}}^{\varrho }, {{\mathcal {I}}_{s}}^{\varrho }, {{\mathcal {I}}_{r}}^{\varrho }, {\mathcal {Q}}^{\varrho }, {\mathcal {R}}^{\varrho })\nonumber \\{} & {} \times \, ((q+1-\varrho )^{\propto }(q-\varrho +2+\propto )-(q-\varrho )^{\propto } (p-\varrho +2+2\propto ))\nonumber \\{} & {} - \, t_{\varrho -1}^{\sigma -1} \Theta _{2}(t_{\varrho },{\mathcal {S}}^{\varrho -1}, {{\mathcal {I}}_{s}}^{\varrho -1}, {{\mathcal {I}}_{r}}^{\varrho -1}, {\mathcal {Q}}^{\varrho -1}, {\mathcal {R}}^{\varrho -1})\nonumber \\{} & {} \times \, ((q-\varrho +1)^{\propto +1}-(p-\varrho )^{\propto }(p-\varrho +1+\propto )) \Big ],\nonumber \\ {{\mathcal {I}}_{r}}^{n+1}(t)= & {} \, {{\mathcal {I}}_{r}}^{0}+ \frac{\sigma t_{n}^{\sigma -1}(1-\propto ) \Theta _{3}(t_{n},{\mathcal {S}}^{n}, {{\mathcal {I}}_{s}}^{n}, {{\mathcal {I}}_{r}}^{n}, {\mathcal {Q}}^{n}, {\mathcal {R}}^{n})}{{\textbf{N}}(\propto )}\nonumber \\{} & {} + \, \frac{\sigma ({\textbf{Q}} t)^{\propto }}{{\textbf{N}}(\propto )\Gamma (\propto +2)}\sum _{\varrho =0}^{q} \Big [t_{\varrho }^{\sigma -1}\Theta _{3}(t_{\varrho },{\mathcal {S}}^{\varrho }, {{\mathcal {I}}_{s}}^{\varrho }, {{\mathcal {I}}_{r}}^{\varrho }, {\mathcal {Q}}^{\varrho }, {\mathcal {R}}^{\varrho })\nonumber \\{} & {} \times \, ((q+1-\varrho )^{\propto }(q-\varrho +2+\propto )-(q-\varrho )^{\propto }(p-\varrho +2+2\propto ))\nonumber \\{} & {} - \, t_{\varrho -1}^{\sigma -1} \Theta _{3}(t_{\varrho },{\mathcal {S}}^{\varrho -1}, {{\mathcal {I}}_{s}} ^{\varrho -1}, {{\mathcal {I}}_{r}}^{\varrho -1}, {\mathcal {Q}}^{\varrho -1}, {\mathcal {R}}^{\varrho -1})\nonumber \\{} & {} \times \, ((q-\varrho +1)^{\propto +1}-(p-\varrho )^{\propto }(p-\varrho +1+\propto )) \Big ],\nonumber \\ {\mathcal {Q}}^{n+1}(t)= & \, {} {\mathcal {Q}}^{0}+\frac{\sigma t_{n}^{\sigma -1}(1-\propto )\Theta _{4}(t_{n}, {\mathcal {S}}^{n}, {{\mathcal {I}}_{s}}^{n}, {{\mathcal {I}}_{r}}^{n}, {\mathcal {Q}}^{n}, {\mathcal {R}}^{n})}{{\textbf{N}}(\propto )}\nonumber \\{} & {} + \, \frac{\sigma ({\textbf{Q}} t)^{\propto }}{{\textbf{N}}(\propto )\Gamma (\propto +2)}\sum _{\varrho =0}^{q} \Big [t_{\varrho }^{\sigma -1}\Theta _{4}(t_{\varrho },{\mathcal {S}}^{\varrho }, {{\mathcal {I}}_{s}}^{\varrho }, {{\mathcal {I}}_{r}}^{\varrho }, {\mathcal {Q}}^{\varrho }, {\mathcal {R}}^{\varrho })\nonumber \\{} & {} \times \, ((q+1-\varrho )^{\propto }(q-\varrho +2+\propto )-(q-\varrho )^{\propto } (p-\varrho +2+2\propto ))\nonumber \\{} & {} - \, t_{\varrho -1}^{\sigma -1} \Theta _{4}(t_{\varrho },{\mathcal {S}}^{\varrho -1}, {{\mathcal {I}}_{s}}^{\varrho -1}, {{\mathcal {I}}_{r}}^{\varrho -1}, {\mathcal {Q}}^{\varrho -1}, {\mathcal {R}}^{\varrho -1})\nonumber \\{} & {} \times \, ((q-\varrho +1)^{\propto +1}-(p-\varrho )^{\propto }(p-\varrho +1+\propto )) \Big ],\nonumber \\ {\mathcal {R}}^{n+1}(t)= & {} \, {\mathcal {R}}^{0}+\frac{\sigma t^{\sigma -1}(1-\propto )\Theta _{5}(t_{n}, {\mathcal {S}}^{n}, {{\mathcal {I}}_{s}}^{n}, {{\mathcal {I}}_{r}}^{n}, {\mathcal {Q}}^{n}, {\mathcal {R}}^{n})}{{\textbf{N}}(\propto )}\nonumber \\{} & {} + \, \frac{\sigma ({\textbf{Q}} t)^{\propto }}{{\textbf{N}}(\propto )\Gamma (\propto +2)}\sum _{\varrho =0}^{q} \Big [t_{\varrho }^{\sigma -1}\Theta _{5}(t_{\varrho },{\mathcal {S}}^{\varrho }, {{\mathcal {I}}_{s}}^{\varrho }, {{\mathcal {I}}_{r}}^{\varrho }, {\mathcal {Q}}^{\varrho }, {\mathcal {R}}^{\varrho })\nonumber \\{} & {} \times \, ((q+1-\varrho )^{\propto }(q-\varrho +2+\propto )-(q-\varrho )^{\propto } (p-\varrho +2+2\propto ))\nonumber \\{} & {} - \, t_{\varrho -1}^{\sigma -1} \Theta _{5}(t_{\varrho },{\mathcal {S}}^{\varrho -1}, {{\mathcal {I}}_{s}} ^{\varrho -1}, {{\mathcal {I}}_{r}}^{\varrho -1}, {\mathcal {Q}}^{\varrho -1}, {\mathcal {R}}^{\varrho -1})\nonumber \\{} & {} \times \, ((q-\varrho +1)^{\propto +1}-(p-\varrho )^{\propto }(p-\varrho +1+\propto )) \Big ]. \end{aligned}$$

## Graphs discussion

The approximate results of the fractal fractional order TB model were discovered by developing a numerical approach. Several scenarios for the fractal fractional order TB model have been examined. We used the aforesaid iterative technique to show the numerical results of the fractal fractional TB model graphically. We choose appropriate values for constants for the model’s parameters for dynamics observation.

**Figure 5:**
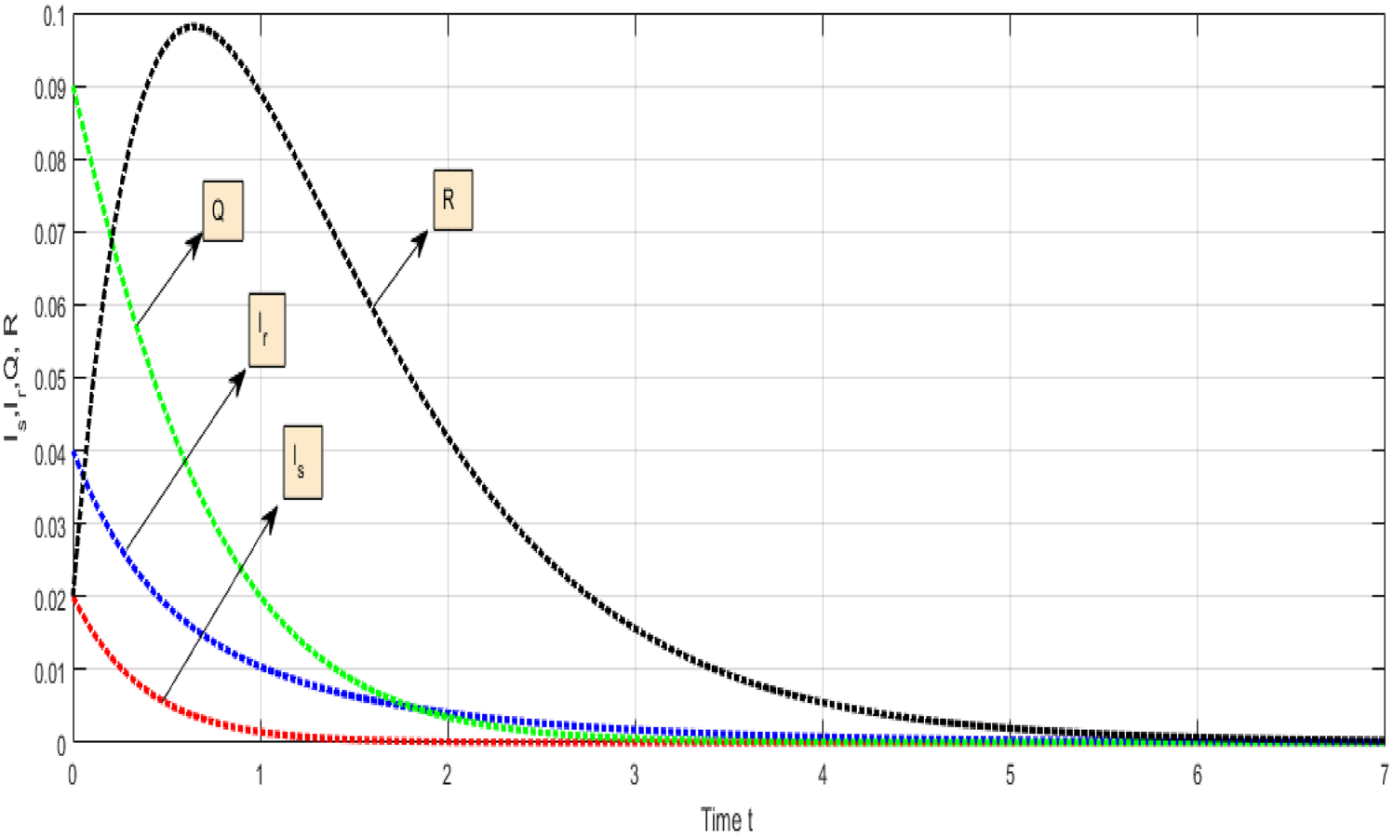
Combine visualization of all class of TB model.

Figure 5 Shows the dynamical representation of combined classes for the integer order value of $\kappa$ and $\propto$ for the non-integer TB model. Considering the settings of the fractional TB model’s parameters such, ${\mathcal {A}}=$ 24,154,133, $\mu =0.007, \theta =0.87, \alpha _1=0.03, \alpha _2=0.14, \alpha 3=0.14, \beta =5.11\times 10^{-10}, \xi =0.035, \sigma =0.5, \theta _1=0.79, \theta _2=0.48, \theta _3=0.48, \gamma =0.42,$ and by considering initial values $({\mathcal {S}}=0.9,{\mathcal {I}}_{s}=0.02,{\mathcal {I}}_{r}=0.04,{\mathcal {Q}}=0.09,{\mathcal {R}}=0.02).$

**Figure 6:**
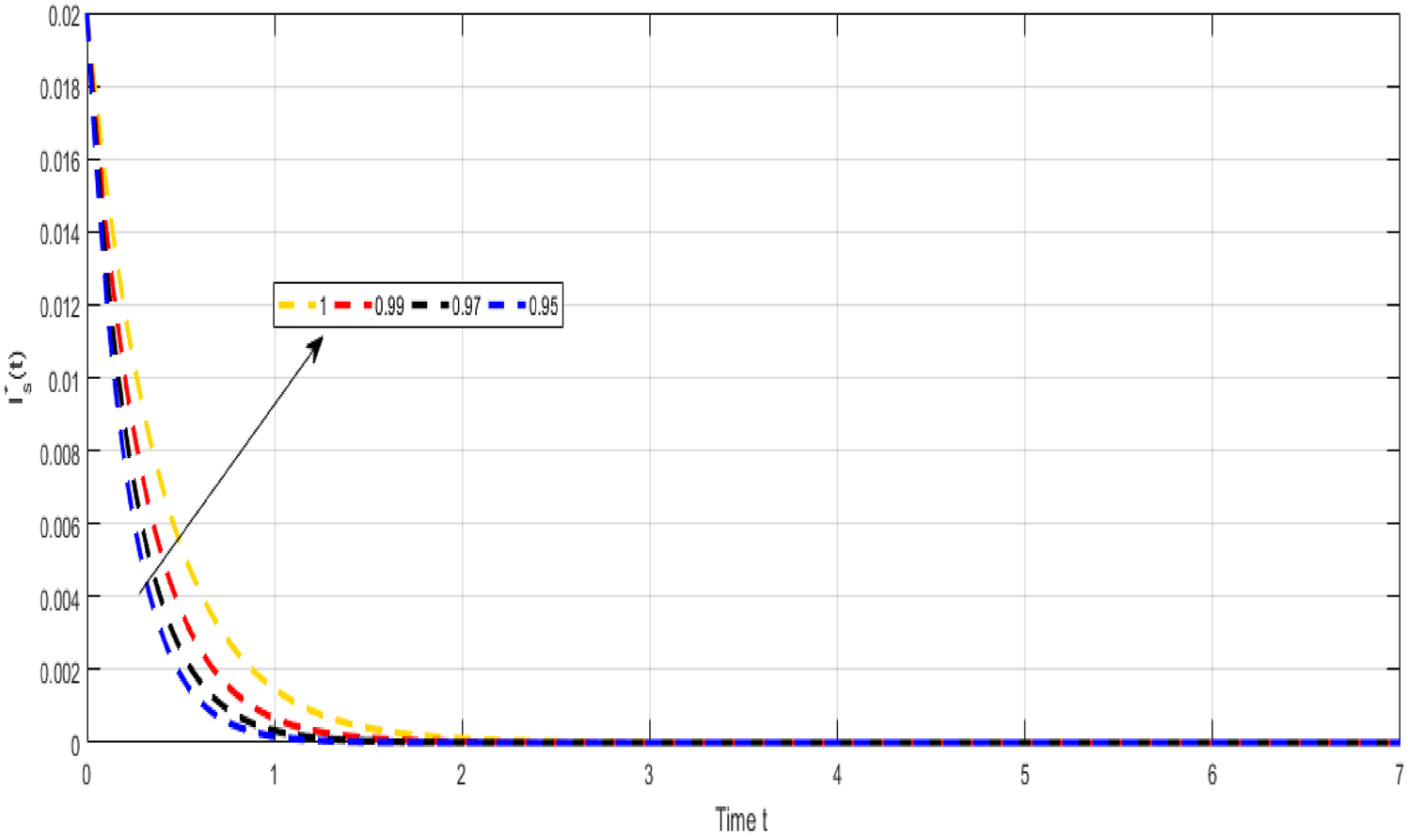
Representation of ${\mathcal {I}}_{s}$ class of TB model.

Figure 6 shows the dynamical visualization of ${\mathcal {I}}_{s}$ class for the different values of $\kappa =\propto =1,0.990,0.970,0.950$ non-integer TB model. Assuming the parameter values in the fractional TB model such, ${\mathcal {A}}=$ 24,154,133, $\mu =0.007, \theta =0.87, \alpha _1=0.03, \alpha _2=0.14, \alpha 3=0.14, \beta =5.11\times 10^{-10}, \xi =0.035, \sigma =0.5, \theta _1=0.79, \theta _2=0.48, \theta _3=0.48, \gamma =0.42,$ and by considering initial values $({\mathcal {S}}=0.9,{\mathcal {I}}_{s}=0.02,{\mathcal {I}}_{r}=0.04,{\mathcal {Q}}=0.09,{\mathcal {R}}=0.02).$

**Figure 7:**
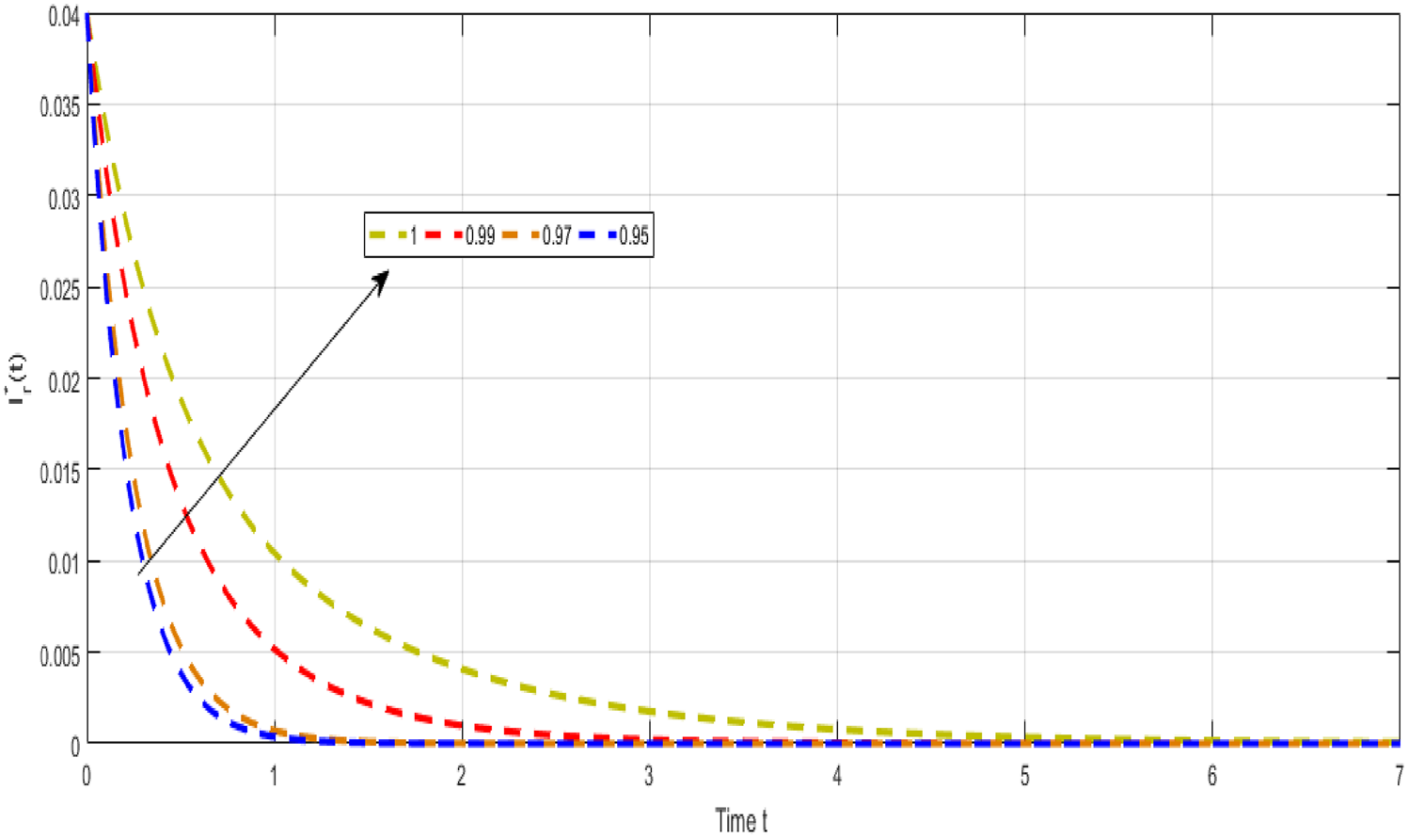
Representation of ${\mathcal {I}}_{r}$ class of TB model.

Figure 7 shows the dynamical visualization of ${\mathcal {I}}_{r}$ class for the different values of $\kappa =\propto =1,0.990,0.970,0.950$ non-integer TB model. Considering the settings of the fractional TB model’s parameters such, ${\mathcal {A}}=$ 24,154,133$, \mu =0.007, \theta =0.87, \alpha _1=0.03, \alpha _2=0.14, \alpha 3=0.14, \beta =5.11\times 10^{-10}, \xi =0.035, \sigma =0.5, \theta _1=0.79, \theta _2=0.48, \theta _3=0.48, \gamma =0.42,$ and by considering initial values $({\mathcal {S}}=0.9,{\mathcal {I}}_{s}=0.02,{\mathcal {I}}_{r}=0.04,{\mathcal {Q}}=0.09,{\mathcal {R}}=0.02).$

**Figure 8:**
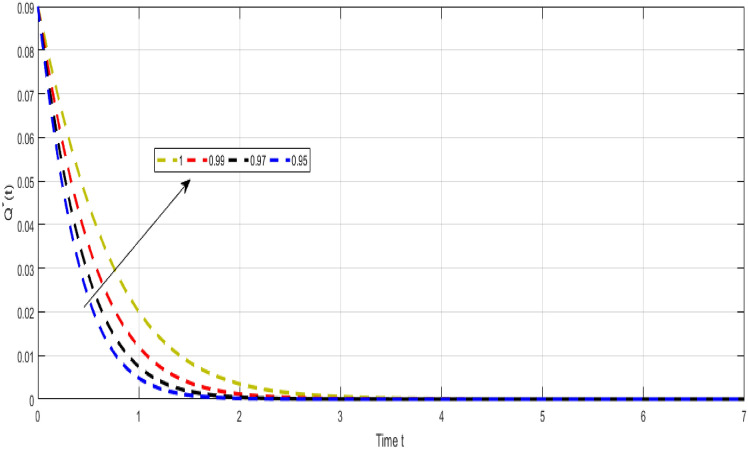
Representation of ${\mathcal {Q}}$ class of TB model.

Figure 8 Shows the dynamical visualization of ${\mathcal {Q}}$ class for the different values of $\kappa =\propto =1,0.990,0.970,0.950$ non-integer TB model. Considering the settings of the fractional TB model’s parameters such, ${\mathcal {A}}=$ 24,154,133, $\mu =0.007, \theta =0.87, \alpha _1=0.03, \alpha _2=0.14, \alpha 3=0.14, \beta =5.11\times 10^{-10}, \xi =0.035, \sigma =0.5, \theta _1=0.79, \theta _2=0.48, \theta _3=0.48, \gamma =0.42,$ and by considering initial values $({\mathcal {S}}=0.9,{\mathcal {I}}_{s}=0.02,{\mathcal {I}}_{r}=0.04,{\mathcal {Q}}=0.09,{\mathcal {R}}=0.02).$

**Figure 9:**
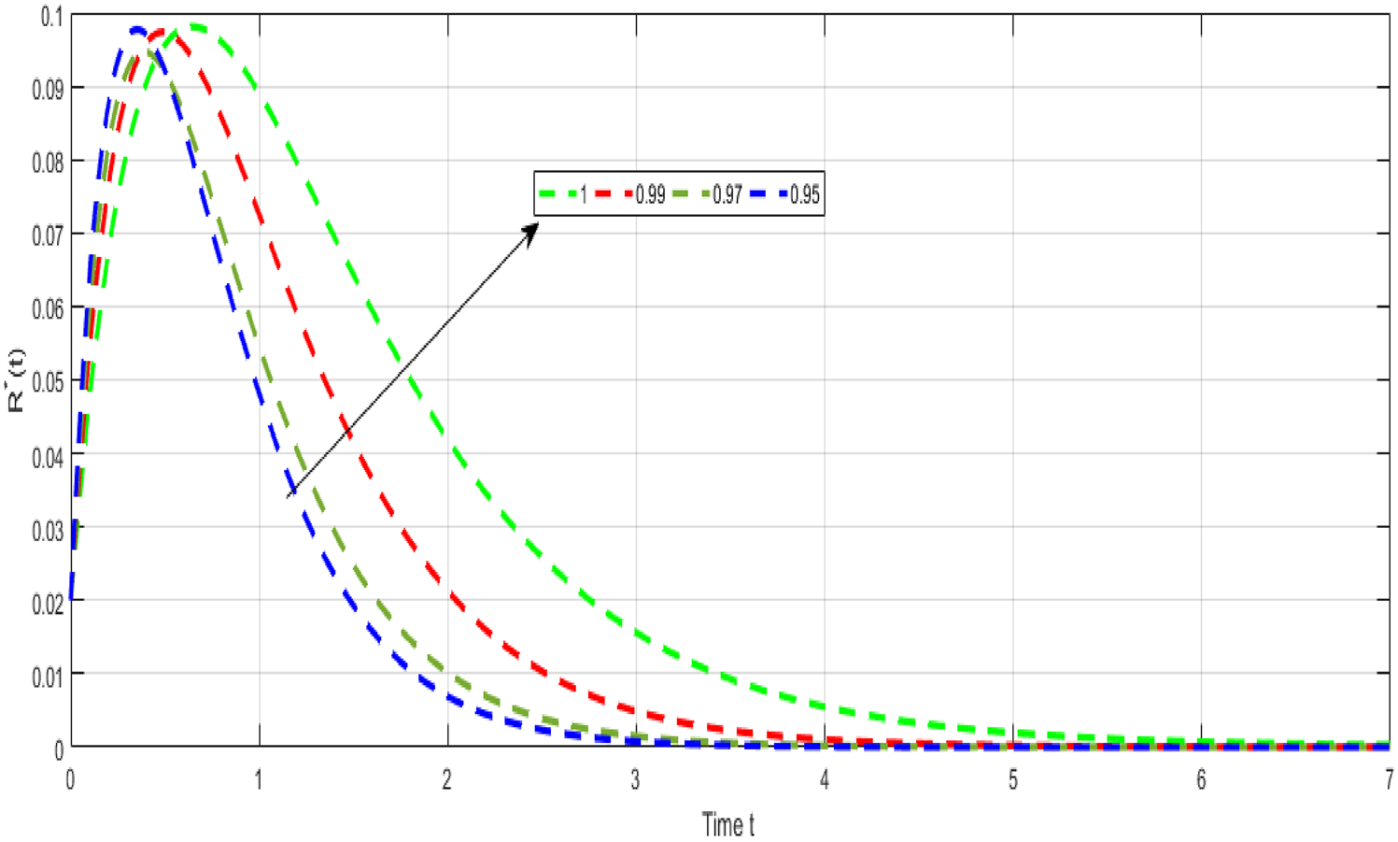
Representation of ${\mathcal {R}}$ class of TB model.

Figure 9 Shows the dynamical visualization of ${\mathcal {R}}$ class for the different values of $\kappa =\propto =1,0.990,0.970,0.950$ non-integer TB model. Considering the settings of the fractional TB model’s parameters such, ${\mathcal {A}}=$ 24,154,133, $\mu =0.007, \theta =0.87, \alpha _1=0.03, \alpha _2=0.14, \alpha 3=0.14, \beta =5.11\times 10^{-10}, \xi =0.035, \sigma =0.5, \theta _1=0.79, \theta _2=0.48, \theta _3=0.48, \gamma =0.42,$ and by considering initial values $({\mathcal {S}}=0.9,{\mathcal {I}}_{s}=0.02,{\mathcal {I}}_{r}=0.04,{\mathcal {Q}}=0.09,{\mathcal {R}}=0.02).$

**Figure 10:**
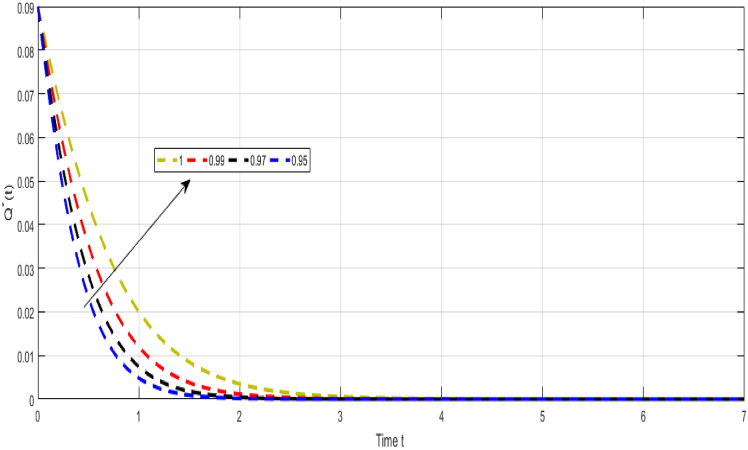
Representation of ${\mathcal {Q}}$ class of TB model.

Figure 10 Shows the dynamical visualization of ${\mathcal {Q}}$ class for the different values of $\kappa =\propto =1,0.990,0.970,0.950$ non-integer TB model. Considering the settings of the fractional TB model’s parameters such, ${\mathcal {A}}=$ 24,154,133, $\mu =0.007, \theta =0.87, \alpha _1=0.03, \alpha _2=0.14, \alpha 3=0.14, \beta =5.11\times 10^{-10}, \xi =0.035, \sigma =0.5, \theta _1=0.79, \theta _2=0.48, \theta _3=0.48, \gamma =0.42,$ and by considering initial values $({\mathcal {S}}=0.9,{\mathcal {I}}_{s}=0.02,{\mathcal {I}}_{r}=0.04,{\mathcal {Q}}=0.09,{\mathcal {R}}=0.02).$

## Conclusion

Despite the abundance of numerical techniques, the significant issues in applied mathematics and engineering have long been the creation of new efficient numerical approaches. The prospect of new numerical approaches for discovering new facts about real-world situations is one of the most compelling incentives for their extensive attention. This work suggests several rapid approximation approaches for solving chaotic structures that employ fractal-fractional operators with novel definitions. The fundamental aspect of these operators is the idea of memory. The course of events shown by such systems may be predicted more correctly with this essential characteristic. Our study reveals a novel model that includes host cell interactions with Mycobacterium tuberculosis into account when describing the dynamics of TB infection in vivo. We implemented fractal fractional operators to enhance validity. We examine chaotic patterns and TB infection solution connections employing computational approaches. Several results assisted in the analysis of key parameters related to system behavior and illustrated the noticeable influence of fractional parameters on results. We explore a strong connection between oscillatory trends and chaos. Numerical results also illuminate the impact of input factors. Further studies endeavor to improve the model by implementing delay and evaluating the impact of vaccination and therapeutic approaches on immune system recuperation and the reduction of tuberculosis symptoms. The proposed innovative strategies were evaluated in the system of non-integer TB model (32). Figure 6 Shows the dynamical visualization of ${\mathcal {I}}_{s}$ class for the different values of $\kappa =\propto =1,0.990,0.970,0.950$ non-integer TB model. Considering the settings of the fractional TB model’s parameters such, ${\mathcal {A}}=$ 24,154,133, $\mu =0.007, \theta =0.87, \alpha _1=0.03, \alpha _2=0.14, \alpha 3=0.14, \beta =5.11\times 10^{-10}, \xi =0.035, \sigma =0.5, \theta _1=0.79, \theta _2=0.48, \theta _3=0.48, \gamma =0.42,$ and by considering initial values $({\mathcal {S}}=0.9,{\mathcal {I}}_{s}=0.02,{\mathcal {I}}_{r}=0.04,{\mathcal {Q}}=0.09,{\mathcal {R}}=0.02).$ Figure 7 Shows the dynamical visualization of ${\mathcal {I}}_{r}$ class for the different values of $\kappa =\propto =1,0.990,0.970,0.950$ non-integer TB model. Considering the settings of the fractional TB model’s parameters such, ${\mathcal {A}}=$ 24,154,133, $\mu =0.007, \theta =0.87, \alpha _1=0.03, \alpha _2=0.14, \alpha 3=0.14, \beta =5.11\times 10^{-10}, \xi =0.035, \sigma =0.5, \theta _1=0.79, \theta _2=0.48, \theta _3=0.48, \gamma =0.42,$ and by considering initial values $({\mathcal {S}}=0.9,{\mathcal {I}}_{s}=0.02,{\mathcal {I}}_{r}=0.04,{\mathcal {Q}}=0.09,{\mathcal {R}}=0.02).$ Figure 8 Shows the dynamical visualization of ${\mathcal {Q}}$ class for the different values of $\kappa =\propto =1,0.990,0.970,0.950$ for the non-integer TB model. Assuming the parameter values in the non-integer TB model such, ${\mathcal {A}}=$ 24,154,133, $\mu =0.007, \theta =0.87, \alpha _1=0.03, \alpha _2=0.14, \alpha 3=0.14, \beta =5.11\times 10^{-10}, \xi =0.035, \sigma =0.5, \theta _1=0.79, \theta _2=0.48, \theta _3=0.48, \gamma =0.42,$ and by considering initial values $({\mathcal {S}}=0.9,{\mathcal {I}}_{s}=0.02,{\mathcal {I}}_{r}=0.04,{\mathcal {Q}}=0.09,{\mathcal {R}}=0.02).$

Figure 9 Shows the dynamical visualization of ${\mathcal {R}}$ class for the different values of $\kappa =\propto =1,0.990$, 0.970, 0.950 non-integer TB model. Assuming the parameter values in the non-integer TB model such, ${\mathcal {A}}=$ 24,154,133, $\mu =0.007, \theta =0.87, \alpha _1=0.03, \alpha _2=0.14, \alpha 3=0.14, \beta =5.11\times 10^{-10}, \xi =0.035, \sigma =0.5, \theta _1=0.79, \theta _2=0.48, \theta _3=0.48, \gamma =0.42,$ and by considering initial values $({\mathcal {S}}=0.9,{\mathcal {I}}_{s}=0.02,{\mathcal {I}}_{r}=0.04,{\mathcal {Q}}=0.09,{\mathcal {R}}=0.02).$ Figure 10 Shows the dynamical visualization of ${\mathcal {Q}}$ class for the different values of $\kappa =\propto =1,0.990,0.970,0.950$ non-integer order TB model. Assuming the parameter values in the non-integer TB model such, ${\mathcal {A}}=$ 24,154,133, $\mu =0.007, \theta =0.87, \alpha _1=0.03, \alpha _2=0.14, \alpha 3=0.14, \beta =5.11\times 10^{-10}, \xi =0.035, \sigma =0.5, \theta _1=0.79, \theta _2=0.48, \theta _3=0.48, \gamma =0.42,$ and by considering initial values $({\mathcal {S}}=0.9,{\mathcal {I}}_{s}=0.02,{\mathcal {I}}_{r}=0.04,{\mathcal {Q}}=0.09,{\mathcal {R}}=0.02).$ The methodologies, when applied to these problems, showed several surprisingly unexpected behaviors of the system, which had reasonable explanations. Several models can be utilized using the numerical techniques described in this study. Future research should follow these crucial paths since stability, consistency, and convergence should be evaluated for any new numerical method.

## Data Availability

All data that support the findings of this study are included in the article.

## References

[1] Ai, J. W., Ruan, Q. L., Liu, Q. H. & Zhang, W. H. Updates on the risk factors for latent tuberculosis reactivation and their managements. *Emerg. Microbes Infect.* **5**(1), 1–8 (2016). 10.1038/emi.2016.10 PMC4777925 26839146

[2] Chakaya, J. *et al.* Global tuberculosis report 2020-reflections on the global TB burden, treatment and prevention efforts. *Int. J. Infect. Dis.* **113**, S7-12 (2021). 33716195 10.1016/j.ijid.2021.02.107 PMC8433257

[3] Fors, J., Strydom, N., Fox, W. S., Keizer, R. J. & Savic, R. M. Mathematical model and tool to explore shorter multi-drug therapy options for active pulmonary tuberculosis. *PLoS Comput. Biol.* **16**(8), e1008107 (2020). 32810158 10.1371/journal.pcbi.1008107 PMC7480878

[4] Awad, S. F., Critchley, J. A. & Abu-Raddad, L. J. Impact of diabetes mellitus on tuberculosis epidemiology in Indonesia: A mathematical modeling analysis. *Tuberculosis* **134**, 102164 (2022). 35288340 10.1016/j.tube.2022.102164

[5] Marimuthu, Y., Nagappa, B., Sharma, N., Basu, S. & Chopra, K. K. COVID-19 and tuberculosis: A mathematical model based forecasting in Delhi, India. *Indian J. Tuberc.* **67**(2), 177–181 (2020). 32553309 10.1016/j.ijtb.2020.05.006 PMC7214306

[6] Ojo, M. M., Peter, O. J., Goufo, E. F., Panigoro, H. S. & Oguntolu, F. A. Mathematical model for control of tuberculosis epidemiology. *J. Appl. Math. Comput.* **69**(1), 69–87 (2023).

[7] Rahman, M. U., Arfan, M., Shah, Z., Kumam, P. & Shutaywi, M. Nonlinear fractional mathematical model of tuberculosis (TB) disease with incomplete treatment under Atangana-Baleanu derivative. *Alex. Eng. J.* **60**(3), 2845–2856 (2021).

[8] Babaei, A., Jafari, H. & Liya, A. Mathematical models of HIV/AIDS and drug addiction in prisons. *Eur. Phys. J. Plus* **135**(5), 1–2 (2020).

[9] Melsew, Y. A. *et al.* Heterogeneous infectiousness in mathematical models of tuberculosis: A systematic review. *Epidemics* **30**, 100374 (2020). 31685416 10.1016/j.epidem.2019.100374

[10] Bhadauria, A. S., Dhungana, H. N., Verma, V., Woodcock, S. & Rai, T. Studying the efficacy of isolation as a control strategy and elimination of tuberculosis in India: A mathematical model. *Infect. Disease Model.* **8**(2), 458–470 (2023). 37234098 10.1016/j.idm.2023.03.005 PMC10206434

[11] Khan, H. *et al.* On a fractal-fractional-based modeling for influenza and its analytical results. *Qual. Theory Dyn. Syst.* **23**(2), 70 (2024).

[12] Ahmed, S., Azar, A. T., Abdel-Aty, M., Khan, H. & Alzabut, J. A nonlinear system of hybrid fractional differential equations with application to fixed time sliding mode control for Leukemia therapy. *Ain Shams Eng. J.* **15**, 102566 (2024).

[13] Khan, H., Alzabut, J., Gómez-Aguilar, J. F. & Alkhazan, A. Essential criteria for existence of solution of a modified-ABC fractional order smoking model. *Ain Shams Eng. J.* **15**, 102646 (2024).

[14] Khan, H. *et al.* A new fractal-fractional hybrid model for studying climate change on coastal ecosystems from the mathematical point of view. *Fractals* 2440015 (2024).

[15] Khan, H., Alzabut, J., Alfwzan, W. F. & Gulzar, H. Nonlinear dynamics of a piecewise modified abc fractional-order leukemia model with symmetric numerical simulations. *Symmetry* **15**(7), 1338 (2023).

[16] Alzabut, J., Dhineshbabu, R., Selvam, A. G., Gómez-Aguilar, J. F. & Khan, H. Existence, uniqueness and synchronization of a fractional tumor growth model in discrete time with numerical results. *Results Phys.* **54**, 107030 (2023).

[17] Zafar, Z. U., Ali, N. & Baleanu, D. Dynamics and numerical investigations of a fractional-order model of toxoplasmosis in the population of human and cats. *Chaos Solitons Fractals.* **151**, 111261 (2021).

[18] Zafar, Z. U., Rehan, K. & Mushtaq, M. HIV/AIDS epidemic fractional-order model. *J. Differ. Equ. Appl.* **23**(7), 1298–1315 (2017).

[19] Zafar, Z. U., Rehan, K., Mushtaq, M. & Rafiq, M. Numerical treatment for nonlinear Brusselator chemical model. *J. Differ. Equ. Appl.* **23**(3), 521–538 (2017).

[20] Zafar, Z. U., Zaib, S., Hussain, M. T., Tunç, C. & Javeed, S. Analysis and numerical simulation of tuberculosis model using different fractional derivatives. *Chaos Solitons Fractals.* **160**, 112202 (2022).

[21] Zafar, Z. U. *et al.* Impact of public health awareness programs on COVID-19 dynamics: A fractional modeling approach. *Fractals* **31**(10), 1–20 (2023).

[22] Zafar, Z. U. *et al.* Numerical simulation and analysis of the stochastic HIV/aids model in fractional order. *Results Phys.* **53**, 106995 (2023).

[23] Zafar, Z. U., Inc, M., Tchier, F. & Akinyemi, L. Stochastic suicide substrate reaction model. *Phys. A* **610**, 128384 (2023).

[24] Shah, K. *et al.* Study of fractional order dynamics of nonlinear mathematical model. *Alex. Eng. J.* **61**(12), 11211–24 (2022).

[25] Khan, H., Alzabut, J., Gómez-Aguilar, J. F. & Agarwal, P. Piecewise mABC fractional derivative with an application. *AIMS Math.* **8**(10), 24345–24366 (2023).

[26] Khan, H., Alam, K., Gulzar, H., Etemad, S. & Rezapour, S. A case study of fractal-fractional tuberculosis model in China: Existence and stability theories along with numerical simulations. *Math. Comput. Simul.* **198**, 455–473 (2022).

[27] Farman, M. *et al.* Fractal fractional-order derivative for HIV/AIDS model with Mittag-Leffler kernel. *Alex. Eng. J.* **61**(12), 10965–80 (2022).

[28] Bonyah, E., Yavuz, M., Baleanu, D. & Kumar, S. A robust study on the listeriosis disease by adopting fractal-fractional operators. *Alex. Eng. J.* **61**(3), 2016–2028 (2022).

[29] Partohaghighi, M. *et al.* A new fractal fractional modeling of the computer viruses system. *Fractals* **30**(05), 2240184 (2022).

[30] Singh, J., Kumar, D. & Baleanu, D. A new analysis of fractional fish farm model associated with Mittag-Leffler-type kernel. *Int. J. Biomath.* **13**(02), 2050010 (2020).

[31] Ben Makhlouf, A. & Baleanu, D. Finite time stability of fractional order systems of neutral type. *Fractal Fract.* **6**(6), 289 (2022).

[32] Baleanu, D., Karaca, Y., Vazquez, L. & Macias-Diaz, J. E. Advanced fractional calculus, differential equations and neural networks: Analysis, modeling and numerical computations. *Phys. Scr.* **98**(11), 110201 (2023).

[33] Partohaghighi, M., Mortezaee, M., Akgül, A., Hassan, A. M. & Sakar, N. Numerical analysis of the fractal-fractional diffusion model of ignition in the combustion process. *Alex. Eng. J.* **86**, 1–8 (2024).

[34] Jan, R., Khan, A., Boulaaras, S. & Ahmed, Zubair S. Dynamical behaviour and chaotic phenomena of HIV infection through fractional calculus. *Discrete Dyn. Nat. Soc.* **2022**, 5937420 (2022).

[35] Jan, R., Boulaaras, S. & Shah, S. A. Fractional-calculus analysis of human immunodeficiency virus and CD4+ T-cells with control interventions. *Commun. Theor. Phys.* **74**(10), 105001 (2022).

[36] Jan, A. *et al.* In vivo HIV dynamics, modeling the interaction of HIV and immune system via non-integer derivatives. *Fractal Fract.* **7**(5), 361 (2023).

[37] Tang, T. Q., Jan, R., Bonyah, E., Shah, Z. & Alzahrani, E. Qualitative analysis of the transmission dynamics of dengue with the effect of memory, reinfection, and vaccination. *Comput. Math. Methods Med.* **2022**, 7893570 (2022). 36238487 10.1155/2022/7893570 PMC9553356

[38] Jan, R., Boulaaras, S., Alnegga, M. & Abdullah, F. A. Fractional-calculus analysis of the dynamics of typhoid fever with the effect of vaccination and carriers. *Int. J. Numer. Model. Electron. Netw. Devices Fields* **37**(2), e3184 (2024).

[39] Tang, T. Q., Shah, Z., Jan, R., Deebani, W. & Shutaywi, M. A robust study to conceptualize the interactions of CD4+ T-cells and human immunodeficiency virus via fractional-calculus. *Phys. Scr.* **96**(12), 125231 (2021).

[40] Kiryakova, V. S. *Generalized Fractional Calculus and Applications* (CRC Press, 1993).

[41] Samko, S. G., Kilbas, A. A., & Marichev, O. I. Integrals and derivatives of the fractional and some of their applications, Nauka i Tehkhnika, Minsk (1987) (**in Russian**).

[42] Podlubny, I. *Fractional Differential Equations* (Academic Press, 1999).

[43] Farman, M., Jamil, S., Nisar, K. S. & Akgul, A. Mathematical study of fractal-fractional leptospirosis disease in human and rodent populations dynamical transmission. *Ain Shams Eng. J.* **15**(3), 102452 (2024).

[44] Kilbas, A. A., Srivastava, H. M. & Trujillo, J. J. *Theory and Applications of Fractional Differential Equations* (Elsevier, 2006).

[45] Paun, M. A., Paun, V. A. & Paun, V. P. Acoustic fractional propagation in terms of porous xerogel and fractal parameters. *Gels* **10**(1), 83 (2024). 38275857 10.3390/gels10010083 PMC10815917

[46] Atangana, A. Fractal-fractional differentiation and integration: Connecting fractal calculus and fractional calculus to predict complex system. *Chaos Solitons Fract.* **102**, 396–406 (2017).

[47] Abdeljawad, T. & Baleanu, D. Discrete fractional differences with nonsingular discrete Mittag-Leffler kernels. *Adv. Differ. Equ.* **2016**(1), 1–22 (2016).

